# Exploring the impact of acetylsalicylic acid and conditioned medium obtained from mesenchymal cells, individually and in combination, on cognitive function, histological changes, and oxidant–antioxidant balance in male rats with hippocampal injury

**DOI:** 10.1002/brb3.70010

**Published:** 2024-09-11

**Authors:** Iman Zangiabadi, Mehran Ilaghi, Ali Shamsara, Seyed Hassan Eftekhar‐Vaghefi, Mona Saheli, Mohammad Shabani

**Affiliations:** ^1^ Department of Anatomy, Afzalipour School of Medicine Kerman University of Medical Sciences Kerman Iran; ^2^ Institute of Neuropharmacology, Kerman Neuroscience Research Center Kerman University of Medical Sciences Kerman Iran

**Keywords:** acetylsalicylic acid, adipose tissue, aspirin, conditioned medium, hippocampus, mesenchymal stem cells

## Abstract

**Background:**

The hippocampus is susceptible to damage, leading to negative impacts on cognition. Conditioned medium (CM) obtained from adipose tissue–derived mesenchymal stem cells (MSCs) and acetylsalicylic acid (ASA) have shown neuroprotective effects independently. This study explored the synergistic potential of ASA and CM from adipose‐derived MSCs against hippocampal injury.

**Methods:**

Adult male Wistar rats received bilateral hippocampal ethidium bromide (EB) injections to induce hippocampal damage. Rats were treated with ASA and/or CM derived from adipose tissue MSCs every 48 h for 16 days. Behavioral tests (open field test, Morris water maze, novel object recognition, and passive avoidance), oxidative stress, Western blot analysis of brain‐derived neurotrophic factor (BDNF) and cerebral dopamine neurotrophic factor (CDNF) expression, and hippocampal histological investigation were conducted.

**Results:**

Administration of EB caused impairments in spatial, recognition, and passive avoidance memory, as well as heightened oxidative stress, reduced BDNF/CDNF expression, and pyramidal cell loss in the hippocampal CA1 region. Administration of ASA, CM, or a combination of both mitigated these hippocampal damages and cognitive deficits, elevated BDNF and CDNF levels, and alleviated the CA1 necrosis caused by EB. Moreover, co‐administering ASA and CM resulted in greater improvements in spatial memory compared to administering ASA alone, suggesting possible synergistic interactions.

**Conclusions:**

The ability of ASA, CM obtained from adipose tissue–derived MSCs, and their combination therapy to alleviate hippocampal injuries highlights their promising therapeutic potential as a neuroprotection strategy against brain damage. Our findings provide preliminary evidence of the potential synergistic effects of ASA and CM, which warrants further investigations.

## Graphical abstract

Acetylsalicylic acid (ASA) and conditioned medium (CM) protect against ethidium bromide–induced hippocampal injury through various mechanisms, including enhancing anti‐inflammatory response, increasing brain neurotrophic factors, and decreasing oxidative stress.

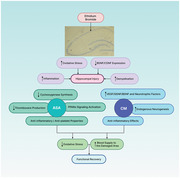

## 1 INTRODUCTION

The hippocampus is a critical region in the mammalian brain, playing a vital role in the consolidation of long‐term memory, spatial navigation, and emotional regulation. Its exceptional plasticity enables it to adapt and respond to various external and internal stimuli (Turner et al., 2022; Zare et al., 2021). However, this plasticity also makes the hippocampus susceptible to injury and damage, which can profoundly affect an individual's cognitive function (Leuner & Gould, 2010; Rajizadeh et al., 2020). Hippocampal injuries, whether due to ischemia, neurodegenerative diseases, or trauma, pose a significant challenge. Therefore, the quest to develop effective strategies to protect and repair this vital region of the brain remains an active and pressing pursuit.

Mesenchymal stem cells (MSCs) represent a category of versatile stem cells that possess the capacity for self‐renewal and the potential to differentiate into various cell types (Eftekhar‐Vaghefi et al., 2015; Han et al., 2019; Mehrabani et al., 2023). Adipose tissue‐derived MSCs are particularly attractive for their ease of isolation and expansion, as well as their remarkable paracrine abilities, which include the secretion of growth factors and anti‐inflammatory mediators (Guillén et al., 2018; Krawczenko & Klimczak, 2022). The conditioned medium (CM) obtained from adipose tissue–derived MSCs is a biologically active environment of these secreted factors and represents a promising avenue in regenerative medicine for promoting tissue repair and reducing inflammation (Stojanović & Najman, 2019; Zhang et al., 2022). Prior research has documented the efficacy of CM obtained from adipose‐derived MSCs in various contexts, including wound healing (Zhang et al., 2022), hair loss (Shin et al., 2015), reversing insulin resistance (Shree & Bhonde, 2017), and immunomodulation (Ivanova‐Todorova et al., 2012). More recently, these CMs have proved to possess neuroprotection (Ramalingam et al., 2022), ameliorate neuroinflammation, and improve hippocampal neuron survival (Mehrabadi et al., 2020), while also showing protection against memory impairments (Akhondzadeh et al., 2020).

On the other hand, acetylsalicylic acid (ASA), commonly referred to as aspirin, is a frequently employed nonsteroidal anti‐inflammatory drug widely consumed as an analgesic or anticoagulant agent. The primary target of aspirin is the enzyme cyclooxygenase. By inhibiting this pro‐inflammatory enzyme, aspirin is renowned for its capacity to reduce the production of prostaglandins. Emerging evidence suggests that ASA could provide neuroprotective effects, particularly in the hippocampal region (Asadi‐Shekaari et al., 2011). For instance, previous studies have shown that ASA possesses the ability to alter hippocampal plasticity (Patel et al., 2018; Pour et al., 2013). Moreover, aspirin could promote hippocampal neurogenesis and working memory (Shamsara et al., 2018; Vergil Andrews et al., 2023), while also holding the capacity to inhibit hippocampal neuronal loss (Ma et al., 2012).

Both CM obtained from adipose‐derived MSCs and ASA have shown neuroprotective effects against hippocampal injuries. However, the combined administration of these agents remains underexplored. This study seeks to investigate the synergistic capability of ASA and CM from adipose tissue–derived MSCs and understand the mechanisms underlying their potential neuroprotective effects.

## 2 MATERIALS AND METHODS

### 2.1 Animals and experimental design

In this study, 128 adult male Wistar rats (250–300 g each) were employed. The animals were kept in a controlled environment with a 12‐h light–dark cycle, maintained at a constant temperature of 25 ± 1°C, and provided unrestricted access to both food and water. The Animal Ethics Committee of Kerman University of Medical Sciences reviewed and granted approval for all procedures involving animal experimentation (IR.KMU.AH.REC.1400.209).

The rats were allocated randomly to eight different experimental groups:

1. Control (CTL)
2. Sham: Received intraperitoneal (IP) injections of saline and bilateral injections into the CA1 region
3. Ethidium bromide (EB): Underwent EB‐induced hippocampal damage
4. EB/CM: Received tail vein injections of 150 μL MSC–CM after EB‐induced hippocampal damage
5. EB/Basal medium (BM): Received tail vein injections of 150 μL BM after EB‐induced hippocampal damage
6. EB/ASA: Received IP injections of 30 mg/kg ASA after EB‐induced hippocampal damage
7. EB/ASA/BM: Received IP injections of 30 mg/kg ASA and 150 μL BM after EB‐induced hippocampal damage
8. EB/ASA/CM: Received IP injections of 30 mg/kg ASA and tail vein injections of 150 μL CM after EB‐induced hippocampal damage.

In all groups receiving drug interventions (ASA, BM, or CM), drugs were injected every 2 days for 16 days (de Cristóbal et al., 2001; Rajizadeh et al., 2019; Tsai et al., 2018).

Behavioral tests were performed on the 8 groups, 16 days after the start of experiments in the following order: On the 16th day of the experiment, the open field test and novel object recognition test were performed. Subsequently, on Days 17 and 18, training sessions and probe trials of the Morris water maze were conducted. Finally, on Days 18 and 19, the passive avoidance learning test was carried out.

**FIGURE 1:**
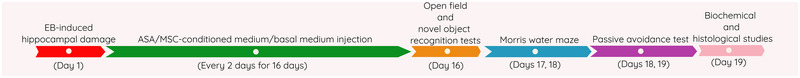
An illustration of the study timeline.

At the end of the experiment, the animals were subjected to deep anesthesia using ketamine (80 mg/kg) and xylazine (10 mg/kg), and their brains were extracted following decapitation. The right hippocampus was isolated and preserved at −80°C until it was processed in the Western blotting analysis for the assessment of brain‐derived neurotrophic factor (BDNF) and cerebral dopamine neurotrophic factor (CDNF) proteins and the determination of oxidative stress markers. Furthermore, the left hippocampus was dissected for histological evaluation. In order to avoid the potential impact of the passive avoidance learning test on oxidative stress markers in this study, we utilized 64 individual rats that had exclusively undergone the drug treatment protocol for groups of our study without any behavioral testing being conducted on them. Figure 1 depicts an illustration of the experimental timeline.

### 2.2 Extraction, cultivation, and expansion of MSCs from adipose tissue

Human subcutaneous adipose tissue was acquired from patients who underwent elective liposuction surgery at the Department of Neurosurgery, Shahid Bahonar Hospital, Kerman, Iran. The procedures for harvesting adipose tissue and preparing human adipose–derived MSCs received approval from the ethical review boards of Kerman University of Medical Sciences. All patients or their authorized representatives provided informed written consent and were informed that the surgery would involve the collection of adipose tissue. The isolation of MSCs was carried out following procedures documented in the existing scientific literature (de Ugarte et al., 2003; Nakagami et al., 2005). Under aseptic conditions, adipose tissues were cut into small pieces before being subjected to digestion in a solution containing 4 mg of Collagenase Type I with a final concentration of 0.1% (Invitrogen Gibco). This digestion process was conducted with gentle agitation for 15 min at 37°C. Following digestion, the mixture was diluted with 4 mL of culture medium, specifically Dulbecco's modified Eagle's medium (DMEM) containing 15% fetal bovine serum (FBS). Subsequently, it was subjected to centrifugation at 1500 revolutions per minute (rpm) for 15 min to segregate the cellular fraction (pellet) from the adipocytes. The supernatant was discarded, and the cellular pellet was subsequently filtered through a 200‐μm nylon mesh to eliminate undigested tissues. Afterward, the isolated cells were cultured in DMEM High Glucose supplemented with 15% FBS (Gibco), along with 100 U/mL of penicillin and 100 μg/mL of streptomycin. These cells were then incubated at a temperature of 37°C in an atmosphere with 5% CO_2_. The initial medium change was carried out approximately 2 days after the start of the culture, during which the non‐adherent cells were removed. Subsequently, this medium change process was repeated every 48–72 h. When the MSCs reached a confluence of 80%–90%, they were subjected to trypsinization using 0.05% trypsin (Sigma) and 0.02% Ethylenediaminetetraacetic acid (EDTA) for the purpose of initiating a new passage. The cells were then cultured until they reached Passage 2 (Akhondzadeh et al., 2020; Shamsara et al., 2018).

### 2.3 Characterization of MSCs through flow cytometry analysis

To verify the extraction of MSCs from human abdominal fat, cell assessment was carried out using flow cytometry. During the second passage, a total of 100,000 cells were marked with specific antibodies, including CD34‐FITC, CD45‐FITC, CD105‐FITC, and CD90‐FITC (Thermo Fisher). These cells were then incubated for 30 min in a refrigerator. Following two washes with phosphate buffered saline (PBS), the cells were fixed in a solution consisting of PBS with 1% paraformaldehyde and 1% FBS. The analysis of the specific fluorescent labeling was conducted using WinMDI 2.9 software (Mehrabadi et al., 2020; Sheibani et al., 2015).

### 2.4 Assessment of MSCs through a differentiation assay

The differentiation capability of MSCs into osteocyte and adipocyte lineages was evaluated during the second passage:

#### 2.4.1 Differentiation of MSCs into osteogenic lineage

MSCs were seeded at a density of 1 × 10^4^ cells per well in 24‐well plates from (SPL) and then incubated at 37°C for 24 h. Subsequently, osteogenic differentiation media, consisting of (100 mM dexamethasone, 10 mM β‐glycerophosphate, and 5 μg/mL ascorbic acid) was added to the cells every 72 h over a period of 3 weeks. The cells were fixed using 4% paraformaldehyde, and the degree of mineralization was assessed by performing Alizarin Red S staining (Akhondzadeh et al., 2020; Shamsara et al., 2018).

#### 2.4.2 Differentiation of MSCs into adipogenic lineage

MSCs were seeded at a concentration of 15 × 10^3 cells per well in 24‐well plates from (SPL) and then incubated at 37°C. After 24 h, adipogenic differentiation media, comprising (100 mM indomethacin, 0.5 mM 3‐isobutyl‐methylxanthine, 250 mM dexamethasone, and 5 mM bovine insulin) was introduced to the cells every 3 days and incubated for a period of 2 weeks. Adipose vacuoles were identified by conducting Oil Red O staining after the cells had been fixed using 4% paraformaldehyde (Eftekhar‐Vaghefi et al., 2015; Akhondzadeh et al., 2020).

### 2.5 Preparation of MSCs’ conditioned medium

CM collection utilized adipose tissue–derived MSCs from the fourth passage. When the cells reached approximately 80% confluence, they were cultured for a duration of 48 h in serum‐free DMEM. Subsequently, the collected conditioned media was subjected to centrifugation at 1800 rpm for 20 min at 4°C to eliminate cell debris. It was then filtered through a 0.22 μm syringe filter (Mehrabadi et al., 2020). Subsequently, supernatants from the MSCs were gathered. The collected media were concentrated by subjecting them to centrifugation at 6000 *g* using an Amicon Ultra‐10 device from (Millipore Corporation) (Egashira et al., 2012). The protein concentration of MSC–CM was determined to be within the range of 700–1400 μg/mL using the Pierce bicinchoninic acid (BCA) protein assay kit (Thermo Fisher Scientific). Following concentration, the media were stored at −80°C for future use.

### 2.6 Hippocampal damage induction

**FIGURE 2:**
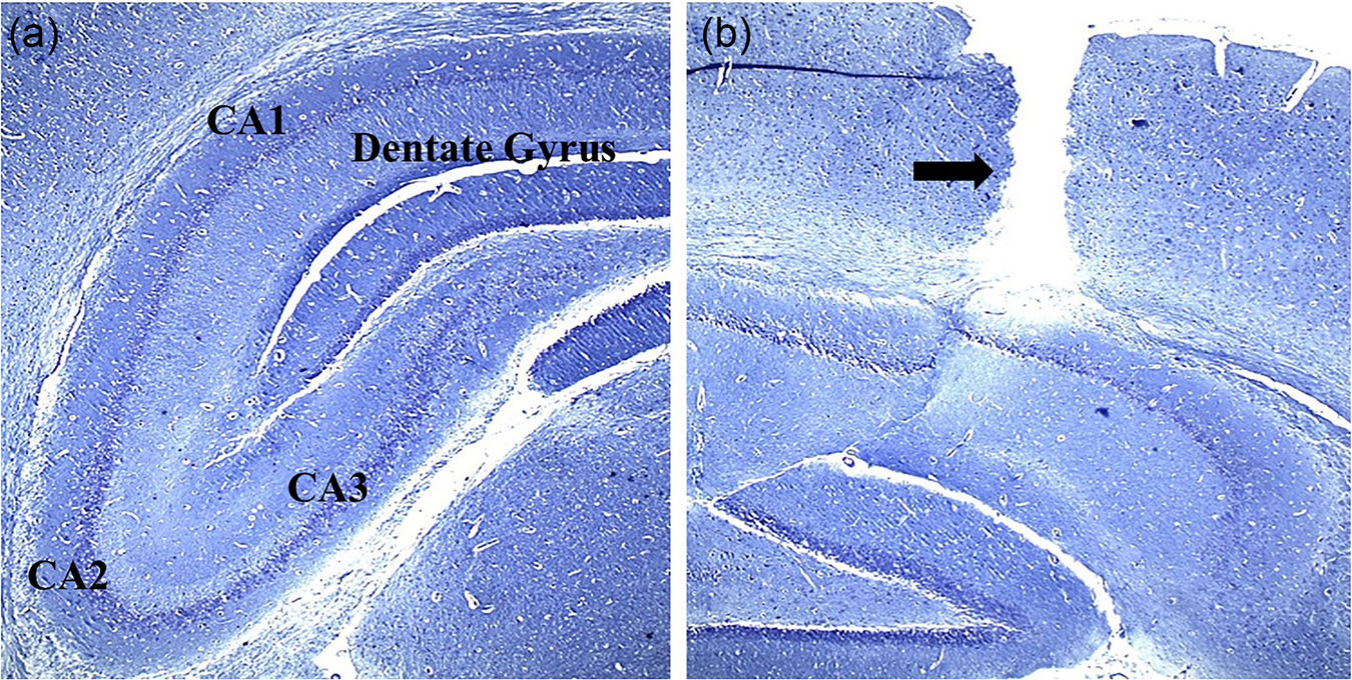
The normal hippocampus regions (a); the needle insertion site (black arrow) for CA1 destruction (10× magnification) (b).

To conduct the surgical procedure for hippocampal damage, the animals underwent deep anesthesia via an IP injection of ketamine (100 mg/kg) and xylazine (20 mg/kg). Subsequently, they were positioned in the skull‐flat orientation on a rat stereotaxic instrument (Stoelting). The hair on the surface of the skull was shaved, and an incision was made to reveal the skull. Two openings were drilled into the skull at precise coordinates to access the CA1 region of the hippocampus. These coordinates were (3.8 mm dorsal to the bregma, 2.4 mm deep from the surface of the dura, and ±2.2 mm laterally) (Hanie et al., 2024). Hippocampal damage in the experimental model was induced bilaterally through a single direct injection of 3 μL of a 0.01% solution of EB from (CinnaGen), into sterile 0.9% saline using a 22‐G needle (Rajizadeh et al., 2019). Figure 2a,b shows the different regions of the hippocampus and the path of the needle throughout the cortex to the hippocampus (Figure 2a,b).

### 2.7 Behavioral tests

After 16 days of the experiment and the administration of drug agents, the rats underwent the following behavioral test. An investigator conducted the test on the animals in a blinded manner, and data were collected using a video image motion analyzer.

#### 2.7.1 Open field test

The open field test is employed to assess anxiety levels, exploratory activity, as well as locomotion in experimental subjects. The open field arena was made of opaque Plexiglas and had dimensions of 90 cm by 90 cm with a height of 45 cm. The arena was divided into 16 small squares, and the amount of time spent in the central and peripheral squares was assessed using a tracking system. The behavior of the rats was recorded and analyzed using an automated video‐tracking system known as the (Noldus EthoVision XT system, version 7). This analysis was conducted at 5‐min intervals. For each rat, the following parameters were recorded and analyzed: the total distance traveled in the center of the arena, the total distance moved, and the number of grooming and rearing behaviors (Shahraki et al., 2022; Zangiabadi et al., 2019).

#### 2.7.2 Novel object recognition test

The novel object recognition test is used to assess nonspatial object memory in rats. In the first phase of this behavioral test, each animal was given the opportunity to explore two similar objects placed in the center of the arena for a duration of 5 min. Following a 45‐min interval, one of the previous similar objects was substituted with a novel object, and the animal's memory retrieval was evaluated during the test phase. During the test phase, each animal was allowed 5 min to discover the novel object. To avoid any instinctual preferences for particular locations, the placement of objects was randomly altered during the first phase of the test. To initiate the tests, rats were positioned between two objects situated in the same location. All the objects used were of similar size but differed in terms of materials and shapes. Between each session, both the location and the objects were cleaned with 70% ethanol to maintain a consistent testing environment. Exploration time was defined as the duration during which the rats sniffed or touched the objects with their snouts. This behavior was recorded using a camera positioned above the maze. The following measurements were recorded: the total time spent exploring the objects during both the first and test phases. Additionally, a discrimination ratio was computed during this test. The discrimination ratio was calculated by subtracting the time spent exploring the familiar object from the time spent exploring the novel object during the second phase. This result was then divided by the total time spent exploring both objects in the second phase (Bejeshk et al., 2023; Zangiabadi et al., 2019).

#### 2.7.3 Morris water maze test

During Days 17 and 18 of the experiment (spanning 2 days), the animals underwent the Morris water maze test to evaluate their spatial learning and memory abilities. The task was conducted between 9:00 a.m. and 1:00 p.m. The apparatus used for this task was a circular pool measuring 140 cm in width and 45 cm in height, filled with water, and maintained at a temperature of 23 ± 2°C. Data were automatically collected using a video image motion analyzer (EthoVision, developed by Noldus Information Technology). In the central area of one quadrant of the pool, a platform measuring 15 cm in width and 35 cm in height was positioned either 1.5 cm above the water's surface (visible) or submerged beneath it. On Day 17 of the visible platform training, the test was conducted. This phase aimed to familiarize the rats with the pool and evaluate their swimming skills and visual capabilities. The hidden platform training, which took place on Days 17 and 18, involved tasks designed to assess the rats’ learning levels. Each day, the rats underwent four trials, with 30‐min intervals between each trial. Rats were placed randomly into the water in one of the four quadrants while facing the wall of the maze. During the acquisition phase, the position of the hidden platform remained constant, and the rats were allotted 60 s to swim to it. On Day 18, following the conclusion of the last training trial, the platform was removed, and a probe trial was conducted to evaluate the extent of memory retention. During the probe trial, the rats were allowed to swim for 60 s, and measurements were taken to assess their retention of spatial memory. This included recording the distance traveled, the time taken to find the target platform (escape latency time), and the amount of time spent in the target quadrant where the platform had been previously located (Abolhasani et al., 2023; Aghaei et al., 2023; Esmaeilpour et al., 2023).

#### 2.7.4 Passive avoidance learning and memory

In this study, the shuttle box apparatus was employed to assess the impact of EB on the passive avoidance learning and memory of rats. The shuttle box consisted of two compartments, one dark and one light, connected by a guillotine door (Rajizadeh et al., 2018; Shabani et al., 2023). The testing phases were conducted over 2 consecutive days as follows:

##### 2.7.4.1 Habituation phase

During the habituation phase, each animal was positioned in the light compartment. After a 10‐s interval, the door separating the two compartments was opened, allowing the animals to freely enter the dark box. The time it took for the rat to exit the light box and enter the dark area was recorded as the step through latency (STL) time. Rats that did not enter the dark compartment within 60 s, likely due to their low motivation to leave the light compartment and their natural inclination to enter the dark area, were excluded from the behavioral test.

##### 2.7.4.2 Training trial phase

Two hours after the habituation phase, the training phase was conducted. Similar to the habituation phase, the animal was initially placed in the light compartment during the training phase. Once the rat entered the dark area during the training phase, an electric shock was administered to the animal's feet and hands. The shock parameters were as follows: frequency 50 Hz, intensity 0.5 mA, and duration 2 s. After receiving the electric shock, the subject was placed back into its cage for a period of 20 s. Following this, after a 2‐min interval, the animal was once again positioned in the light compartment. Upon reentry into the dark compartment, the animal was subjected to another electric shock. The count of these shocks was used as an index of learning. The maximum cut‐off time for STL was set at 300 s. If a rat took longer than this time to enter the dark compartment during the training phase, it would not receive another shock, and its time would be recorded as 300 s.

##### 2.7.4.3 Retention trial phase

Long‐term memory retention was assessed 24 h after the training trial. During this retention trial phase, the animal was placed in the light compartment, and the latency to enter the dark compartment was recorded. During this phase, no shock was administered to the animal. The maximum cut‐off time for STL was set at 300 s (Zangiabadi et al., 2019).

### 2.8 Assessment of hippocampal oxidative stress markers

After completing the behavioral tests, the rats were deeply anesthetized through IP injections of ketamine (80 mg/kg) and xylazine (10 mg/kg). After making sure the animal was anesthetized, it was sacrificed by the cervical dislocation method. The skin and muscles of the neck and head were dissected. Afterward, using a special guillotine, the animal's head was separated. The scalp was cut with scissors along the sagittal suture. The muscles were removed. By placing the tip of the scissors in the foramen magnum and repeating this procedure from the nasal side, the brain and meninges were slowly exposed. The removal of the meninges was done carefully. Following the removal of the brain, the hippocampal tissue was dissected and subsequently homogenized in cold PBS (with a pH of 7.4). The specimens were subjected to centrifugation at 10,000 times the force of gravity (at 4°C). Afterward, the resulting supernatants were gathered and preserved at −80°C until they were ready for subsequent examination.

The concentration of malondialdehyde (MDA) was determined using the thiobarbituric acid reactive substance method (Zarin et al., 2019). Superoxide dismutase (SOD) and total antioxidant capacity (TAC) levels were measured using commercially available assay kits (KIAZIST Life Sciences) employing the colorimetric method. To determine the protein content in the tissue, the BCA Protein Quantification Kit (Parstous) was employed (Aghaei et al., 2023; Rajizadeh et al., 2023).

### 2.9 Western blot analysis

Hippocampus tissue specimens were homogenized with the use of a lysis buffer, and subsequently, total protein extract was obtained through centrifugation at 15,000 rpm for 5 min. The protein content in the resulting supernatants was quantified using Bradford's method. Lysates containing 60 μg of protein each were loaded onto a 12% SDS–PAGE gel and subsequently transferred to a polyvinylidene difluoride (PVDF) membrane (Chemicon Millipore Co.). The membranes were blocked using a 2% blocking reagent from the Electrochemiluminescence Advanced Kit (Amersham Bioscience Co.) and were then incubated separately with primary antibodies overnight. Following three washes with Tris‐buffered saline with Tween 20, the blots were subjected to incubation with a secondary antibody, specifically rabbit IgG horseradish peroxidase conjugated (dilution of 1/3000, Cell Signaling Technology Co.), for a duration of 1 h at room temperature. Additionally, the bands that displayed reactivity were identified using a chemiluminescence kit reagent (Amersham Bioscience Co.). Quantification of the blots was carried out using the ImageJ software. Rabbit anti‐β‐Actin (dilution of 1/1000, Cell Signaling Technology Co.) served as the internal control (Akhondzadeh et al., 2020; Bejeshk et al., 2023).

### 2.10 Histological studies

Following the completion of behavioral tests, the animals were subjected to deep anesthesia by administering a combination of ketamine (at a dose of 80 mg/kg) and xylazine (at a dose of 10 mg/kg). After cutting the olfactory bulb, the brain was removed from the skull cavity. An incision about 3–4 mm from the frontal pole was made with a razor to separate the frontal cortex and hippocampus. The left hippocampus was meticulously extracted and then placed in a 10% formalin solution for preservation. The specimens were cut into sections after being embedded in optimal cutting temperature using cryomolds. Sequential coronal sections of the hippocampus, each measuring 7 μm in thickness, were meticulously sliced using a cryostat machine (SLEE). These sections were then preserved in a cryoprotectant solution at −20°C until they were ready to be utilized for Cresyl violet staining. A uniform systematic random sampling approach was employed, where every seventh section, with intervals of 350 μm, was placed onto slides coated with gelatin. These slides were then left at room temperature overnight. In each group, 100 fields on the slides were chosen at random. The selection of fields was done by matching them between the two groups based on the adult rat brain atlas (Watson, 2007). The samples were examined using an optical microscope set at a magnification of ×400. Only pyramidal neurons located in the CA1 region of the left hippocampus, which displayed a distinct and visible nucleus as well as a nucleolus, were categorized as viable and undamaged cells (Zangiabadi et al., 2019).

### 2.11 Statistical analysis

The data underwent statistical analysis using Prism (Version 8). In order to assess the normality of data distribution, the Shapiro–Wilk test or *K*–*S* test will be employed. For normally distributed data, parametric tests such as repeated‐measures analysis of variance (ANOVA) and one‐way ANOVA with Bonferroni correction were used. Otherwise, the nonparametric Kruskal–Wallis test followed by Dunn's multiple comparisons test was utilized. The data were presented using the mean ± standard error of the mean, and a significance level of *p* < .05 was established as the threshold for statistical significance.

## 3 RESULTS

### 3.1 Demonstration of mesenchymal cells

**FIGURE 3:**
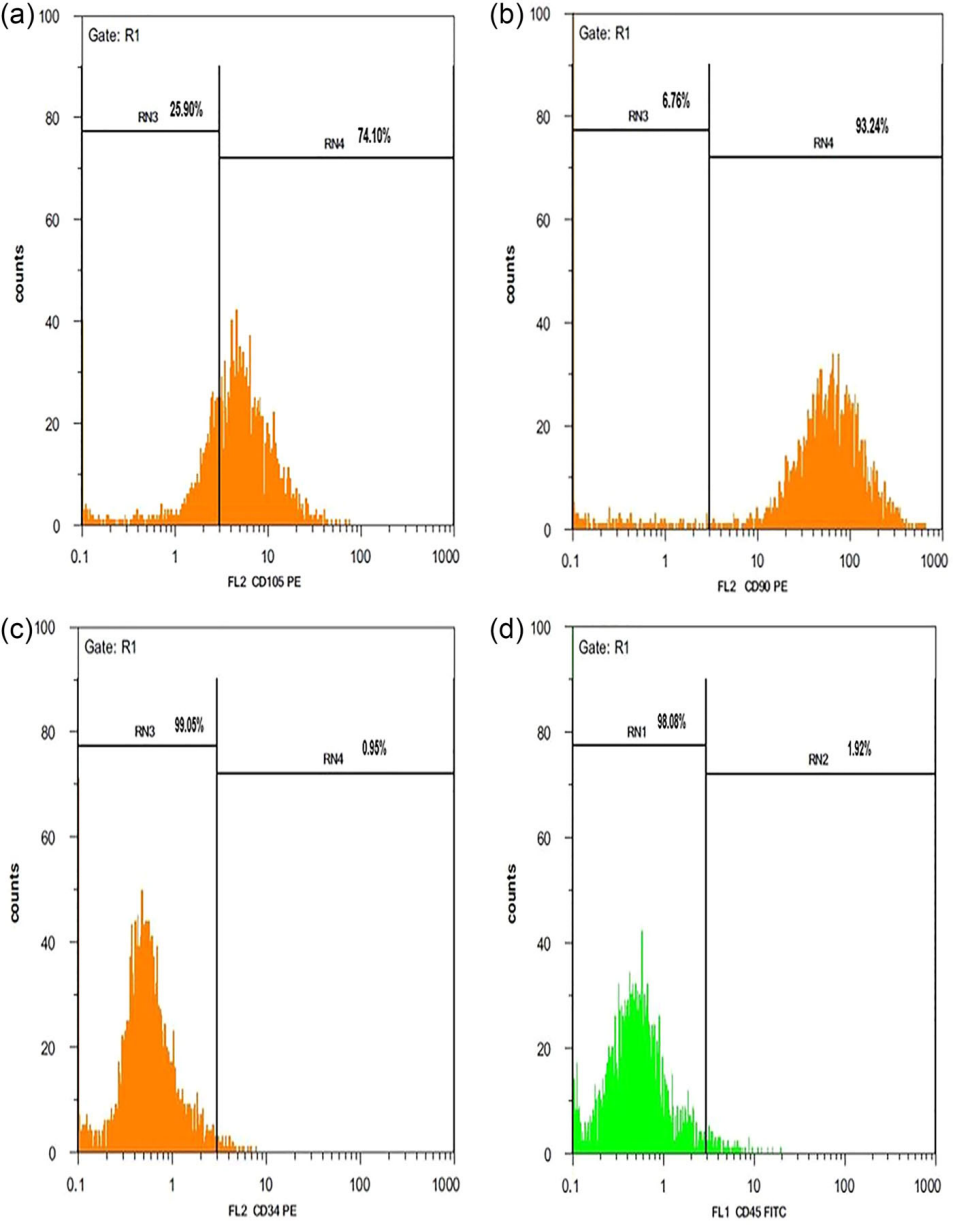
The characterization of mesenchymal stem cells (MSCs) derived from rat adipose tissue was conducted through flow cytometry analysis. MSCs in (a) and (b) exhibited elevated expression levels of mesenchymal markers (CD105 and CD90). Parts (c) and (d) displayed lower levels of hematopoietic markers (CD34 and CD45).

**FIGURE 4:**
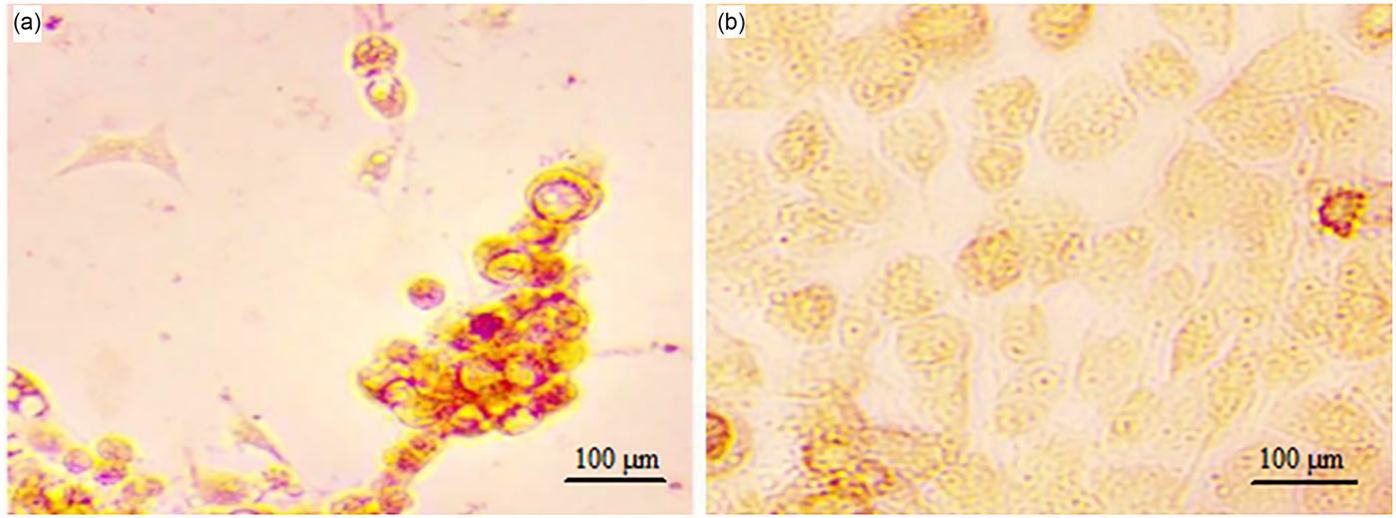
Photomicrographs of the cell cultures derived from mesenchymal stem cells (MSCs) isolated from adipose tissue were taken. During the adipogenic differentiation of the cells, lipid droplets that formed were stained red using the Oil Red staining method (a). In the Alizarin Red staining method, areas of osteogenic activity were visualized as red regions (b).

Confirmation of MSCs derived from adipose tissue was done through flow cytometry analysis. MSCs showed higher mesenchymal markers (CD105 and CD90) and lower hematopoietic markers (CD34 and CD45) (Figure 3). Moreover, to confirm that stem cells are mesenchymal, these cells were differentiated into adipo‐ and osteo‐cells. During the adipogenic differentiation of the cells, the lipid droplets formed were stained red using the Oil Red staining method (Figure 4a). In the Alizarin Red staining method, osteogenic activity areas were observed as red areas (Figure 4b).

### 3.2 Behavioral tests

#### 3.2.1 Morris water maze

**FIGURE 5:**
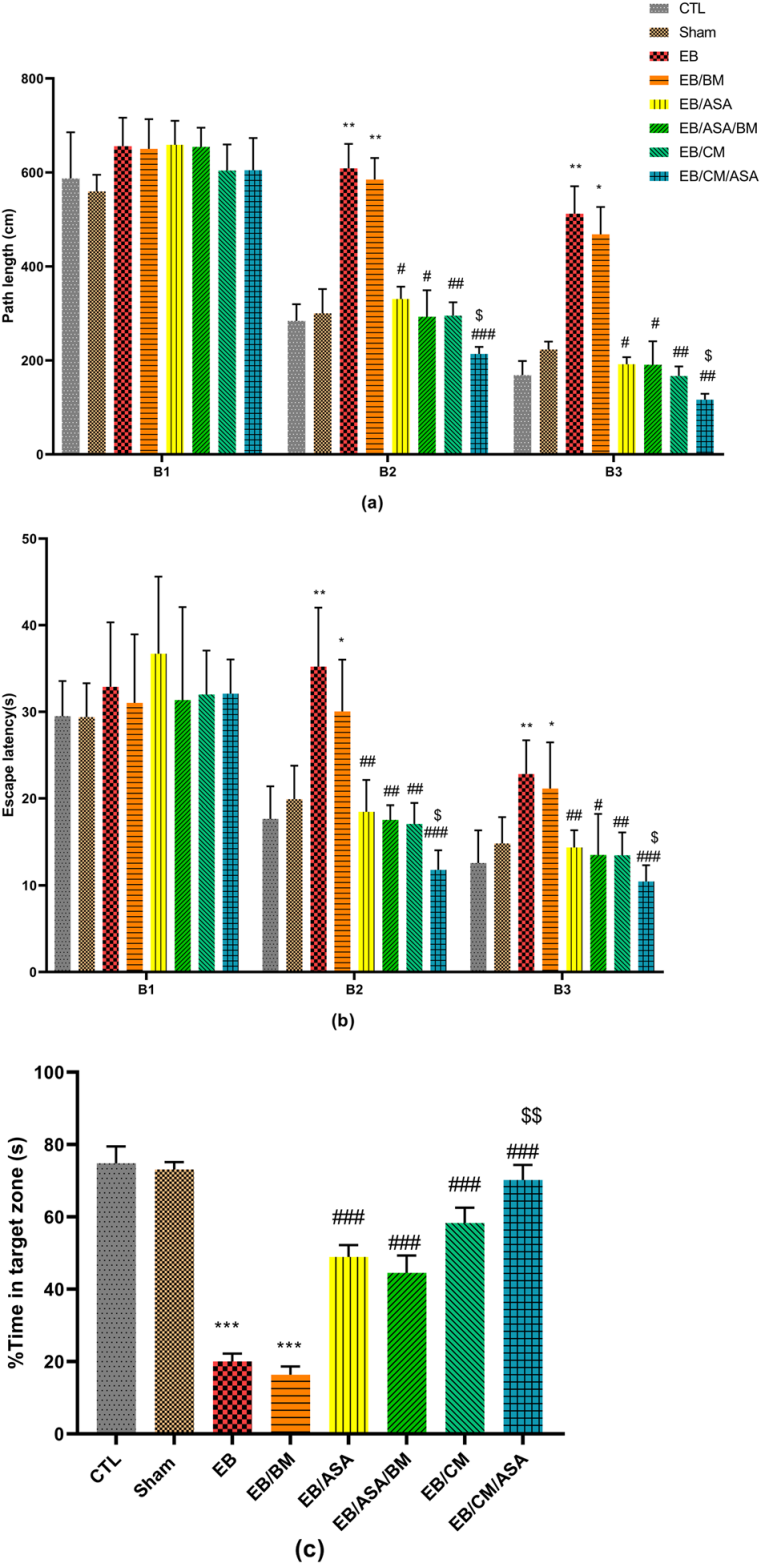
The effects of acetylsalicylic acid (ASA), basal medium (BM), conditioned medium (CM), and local ethidium bromide (EB) injection on spatial learning and memory in Morris water maze (MWM) task: (a) distance traveled to locate the hidden platform; (b) time spent to locate the hidden platform; (c) percentage of distance traveled. Data are expressed as the mean ± standard error of the mean (SEM) (*p < .05, **p < .01, ***p < .001) compared to the control group and (#*p* < .05, ##*p* < .01, ###*p* < .001) compared to the EB group and ($*p* < .05, $$*p* < .01) compared to the EB/ASA group. CTL, control; EB/ASA, ASA‐treated EB group; EB/ASA/BM, BM‐ and ASA‐treated EB group; EB/BM, BM‐treated EB group; EB/CM, mesenchymal stem cell (MSC)–CM‐treated EB group; EB/CM/ASA, CM‐ and ASA‐treated EB group; Sham, sham.

EB group demonstrated a significantly higher traveled distance (Figure 5a) and escape latency time (Figure 5b) in Blocks 2 and 3 for locating the platform over 2 days compared to the equivalent metrics in the control group. Similarly, in EB/BM group, an increase in both the distance traveled and the time taken to locate the hidden platform during Blocks 2 and 3 was observed. Administration of ASA, CM, or co‐administration of CM/ASA resulted in a significant improvement in learning and memory as seen by reduced distance traveled and escape latency in Blocks 2 and 3 compared to the EB group (Figure 5a,b). Notably, the EB/CM/ASA group exhibited a reduction in both the traveled distance and escape latency when compared to the EB/ASA group, suggesting a synergistic effect of ASA and CM (Figure 5a,b).

In the probe trial, it was seen that the percentage of time spent in the target quadrant was significantly lower in both the EB and EB/BM groups when compared to the control group (Figure 5c). On the other hand, the administration of ASA and CM in EB/ASA, EB/ASA/BM, EB/CM, and EB/CM/ASA groups resulted in a significant increase in the percentage of target quadrant time compared to the EB group. Furthermore, the EB/CM/ASA group displayed significant increase in the percentage of target quadrant time compared to the EB/ASA group (Figure 5c).

#### 3.2.2 Open field test

**FIGURE 6:**
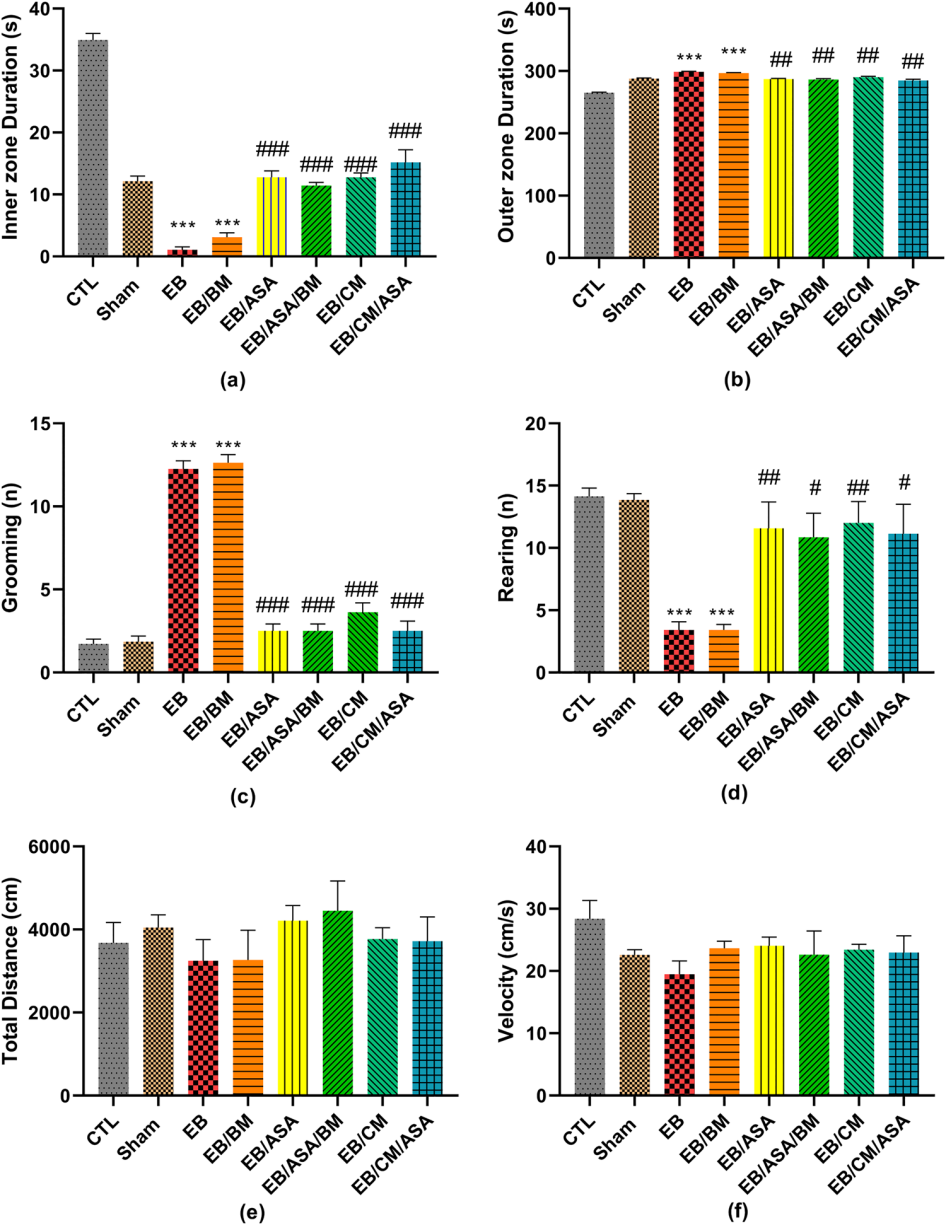
The effects of acetylsalicylic acid (ASA), basal medium (BM), conditioned medium (CM), and local ethidium bromide (EB) injection on an open field test: (a) time spent in the inner zones of box; (b) time spent in the outer zones of box; (c) number of grooming; (d) number of rearing; (e) total distance moved in box; (f) velocity of movement. Data are expressed as the mean ± standard error of the mean (SEM) (***p < .001) compared to the control group and (#*p* < .05, ##*p* < .01, ###*p* < .001) compared to the EB group. CTL, control; EB/ASA, ASA‐treated EB group; EB/ASA/BM, BM‐ and ASA‐treated EB group; EB/BM, BM‐treated EB group; EB/CM, mesenchymal stem cell (MSC)–CM‐treated EB group; EB/CM/ASA, CM‐ and ASA‐treated EB group; Sham, sham.

When compared to the control group, the time spent in the inner zone and the number of rearing were significantly decreased in EB and EB/BM groups (Figure 6a,d). Moreover, the time spent in the outer zone and the number of grooming were significantly increased in these groups compared to the control group (Figure 6b,c). However, EB/ASA, EB/ASA/BM, EB/CM, and EB/CM/ASA groups showed significantly decreased time spent in the periphery and the number of grooming (Figure 6b,c) as well as increased time spent in the central zone and the number of rearing (Figure 6a,d) compared to the EB group. Nevertheless, there were no significant differences in total traveled distance (Figure 6e) and movement velocity (Figure 6f) among the groups.

#### 3.2.3 Novel object recognition

**FIGURE 7:**
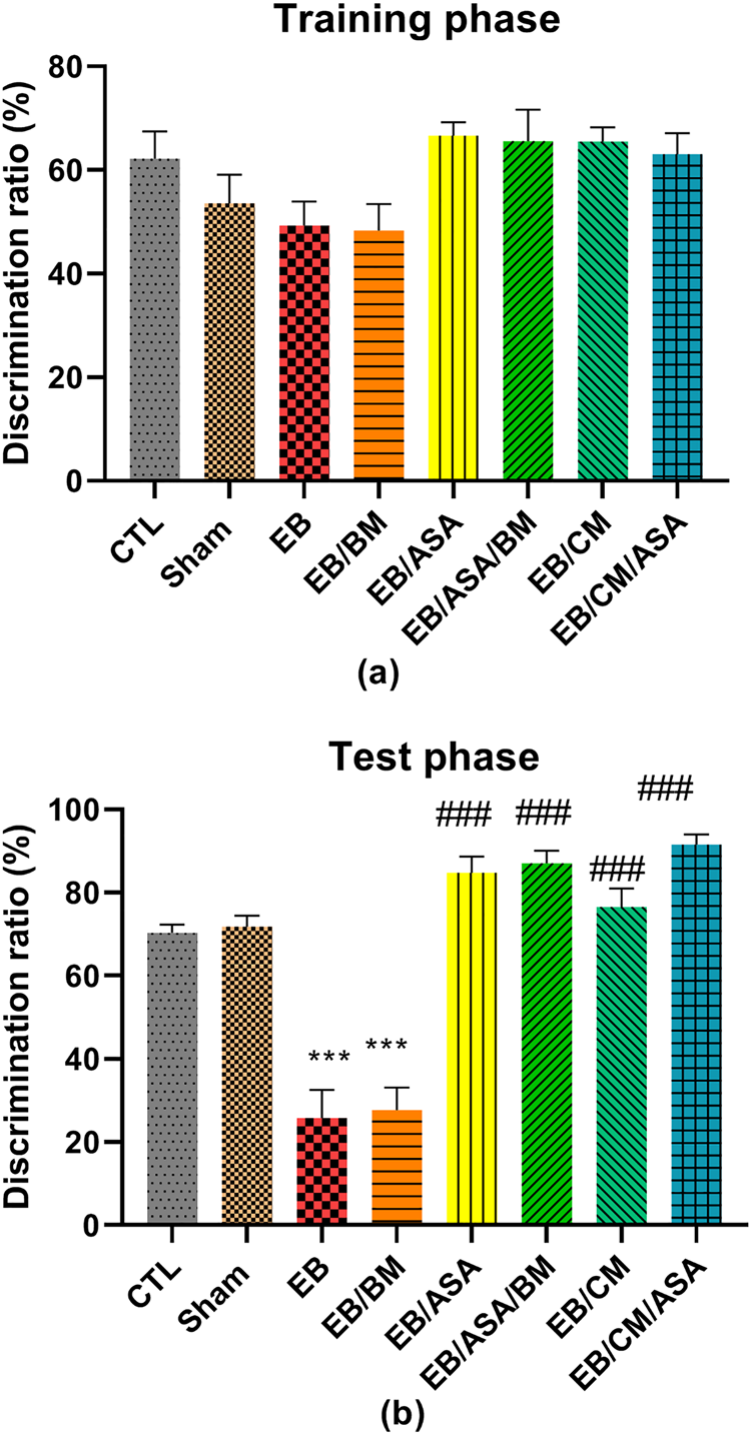
The effects of acetylsalicylic acid (ASA), basal medium (BM), conditioned medium (CM), and local ethidium bromide (EB) injection in the novel object recognition test: (a) discrimination ratio in training phase; (b) discrimination ratio in testing phase. Data are expressed as the mean ± standard error of the mean (SEM) (***p < .001) compared to the control group and (###*p* < .001) compared to the EB group. CTL, control; EB/ASA, ASA‐treated EB group; EB/ASA/BM, BM‐ and ASA‐treated EB group; EB/BM, BM‐treated EB group; EB/CM, mesenchymal stem cell (MSC)–CM‐treated EB group; EB/CM/ASA, CM‐ and ASA‐treated EB group; Sham, sham.

There were no significant differences in discrimination ratio during the training phase of novel object recognition test (Figure 7a). However, EB and EB/BM groups demonstrated significant decreased discrimination ratio during test phase compared with the control group (Figure 7b). Moreover, EB/ASA, EB/ASA/BM, EB/CM, and EB/CM/ASA groups had significantly increased discrimination ratio during test phase compared with the EB group (Figure 7b).

#### 3.2.4 Passive avoidance learning and memory

**FIGURE 8:**
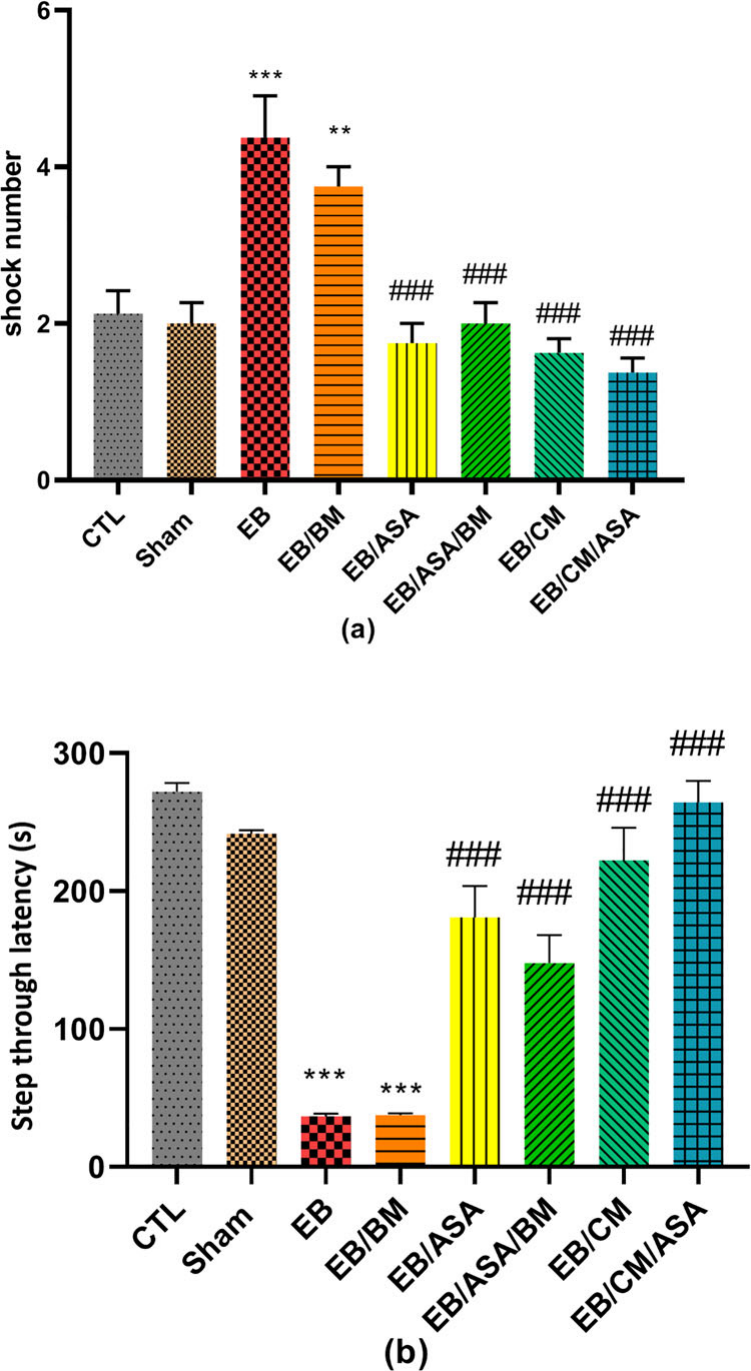
The effects of acetylsalicylic acid (ASA), basal medium (BM), conditioned medium (CM), and local ethidium bromide (EB) injection in the passive avoidance learning test: (a) number of shocks; (b) step through latency to enter the dark compartment. Data are expressed as the mean ± standard error of the mean (SEM) (**p < .01, ***p < .001) compared to the control group and (###*p* < .001) compared to the EB group. CTL, control; EB/ASA, ASA‐treated EB group; EB/ASA/BM, BM‐ and ASA‐treated EB group; EB/BM, BM‐treated EB group; EB/CM, mesenchymal stem cell (MSC)–conditioned medium (CM)‐treated EB group; EB/CM/ASA, CM‐ and ASA‐treated EB group; Sham, sham.

EB and EB/BM groups showed a significantly increased number of shocks compared with control group (Figure 8a), implying impaired passive avoidance learning. However, EB/ASA, EB/ASA/BM, EB/CM, and EB/CM/ASA groups showed a significantly decreased number of shocks compared with the EB group (Figure 8a), suggesting that these agents reversed learning impairments induced by EB. Furthermore, STL was reduced in EB and EB/BM groups compared with the control group (Figure 8b). However, EB/ASA, EB/ASA/BM, EB/CM, and EB/CM/ASA demonstrated significantly less desire toward the dark chamber compared to the EB group, suggesting that ASA and CM have a beneficial effect in alleviating the harmful impact of EB on passive avoidance memory.

### 3.3 Oxidative markers

**FIGURE 9:**
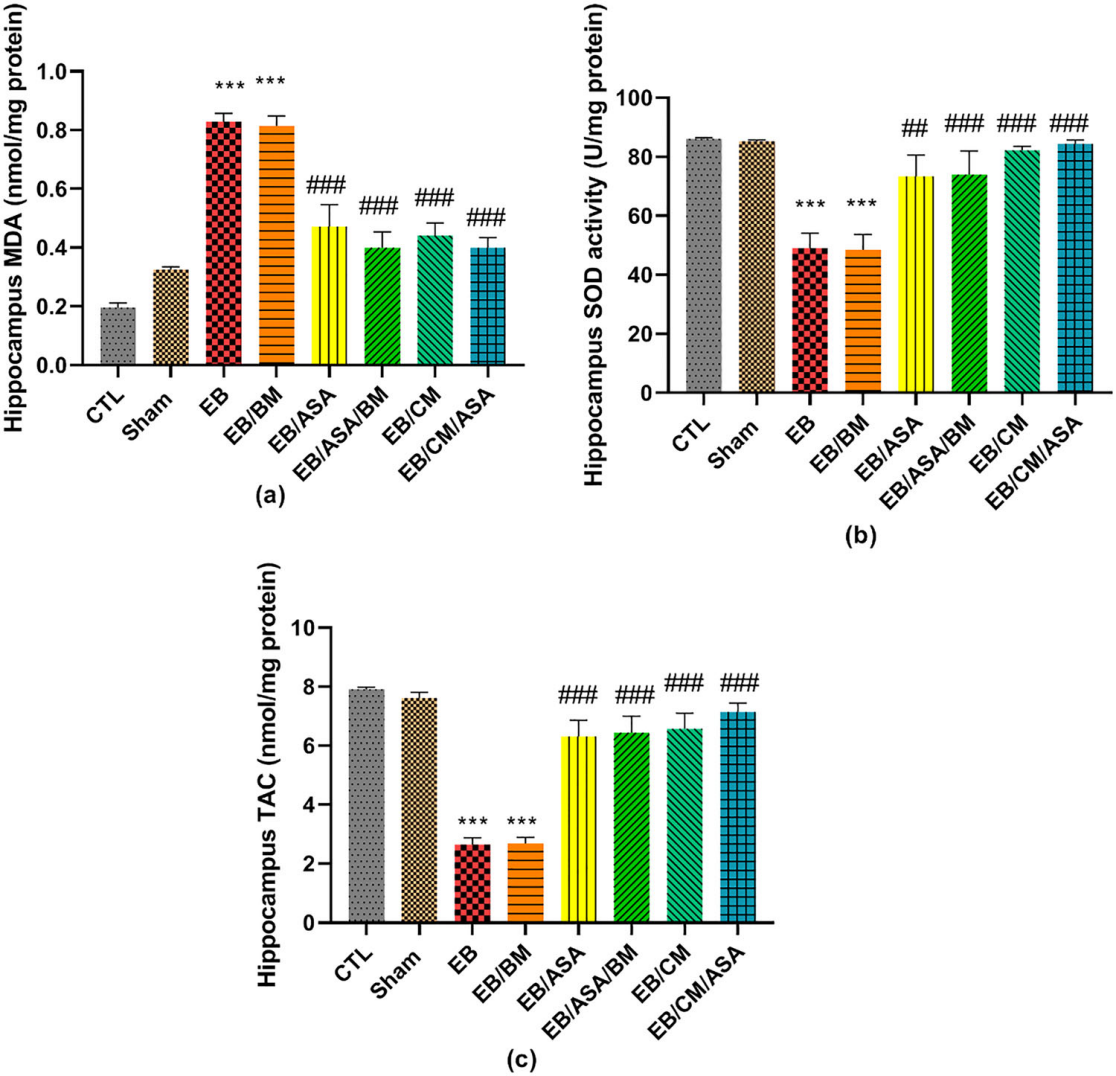
The effects of acetylsalicylic acid (ASA), basal medium (BM), conditioned medium (CM), and local ethidium bromide (EB) injection on hippocampal oxidative markers: (a) malondialdehyde (MDA) ; (b) superoxide dismutase (SOD) activity; (c) total antioxidant capacity (TAC).Data are expressed as the mean ± standard error of the mean (SEM) (***p < .001) compared to the control group and (##*p* < .01, ###*p* < .001) compared to the EB group. CTL, control; EB/ASA, ASA‐treated EB group; EB/ASA/BM, BM‐ and ASA‐treated EB group; EB/BM, BM‐treated EB group; EB/CM, mesenchymal stem cell (MSC)–CM‐treated EB group; EB/CM/ASA, CM‐ and ASA‐treated EB group; Sham, sham.

The hippocampal MDA levels in EB and EB/BM rats were observed to be significantly higher than in the control group (Figure 9a). On the other hand, the MDA concentration was significantly reduced to a level comparable to control group in the EB/ASA, EB/ASA/BM, EB/CM, and EB/CM/ASA groups (Figure 9a). Moreover, the EB and EB/BM groups exhibited reduced SOD antioxidant activity compared to the control group (Figure 9b). Reciprocally, the SOD activity in the EB/ASA, EB/ASA/BM, EB/CM, and EB/CM/ASA groups was significantly higher than in the EB group (Figure 9b). Furthermore, the hippocampal TAC levels were significantly increased following EB and EB/BM administrations when compared to the control group (Figure 9c). However, the TAC levels in EB/ASA, EB/ASA/BM, EB/CM, and EB/CM/ASA groups showed significant increase than the EB group (Figure 9c).

### 3.4 Western blot analysis

**FIGURE 10:**
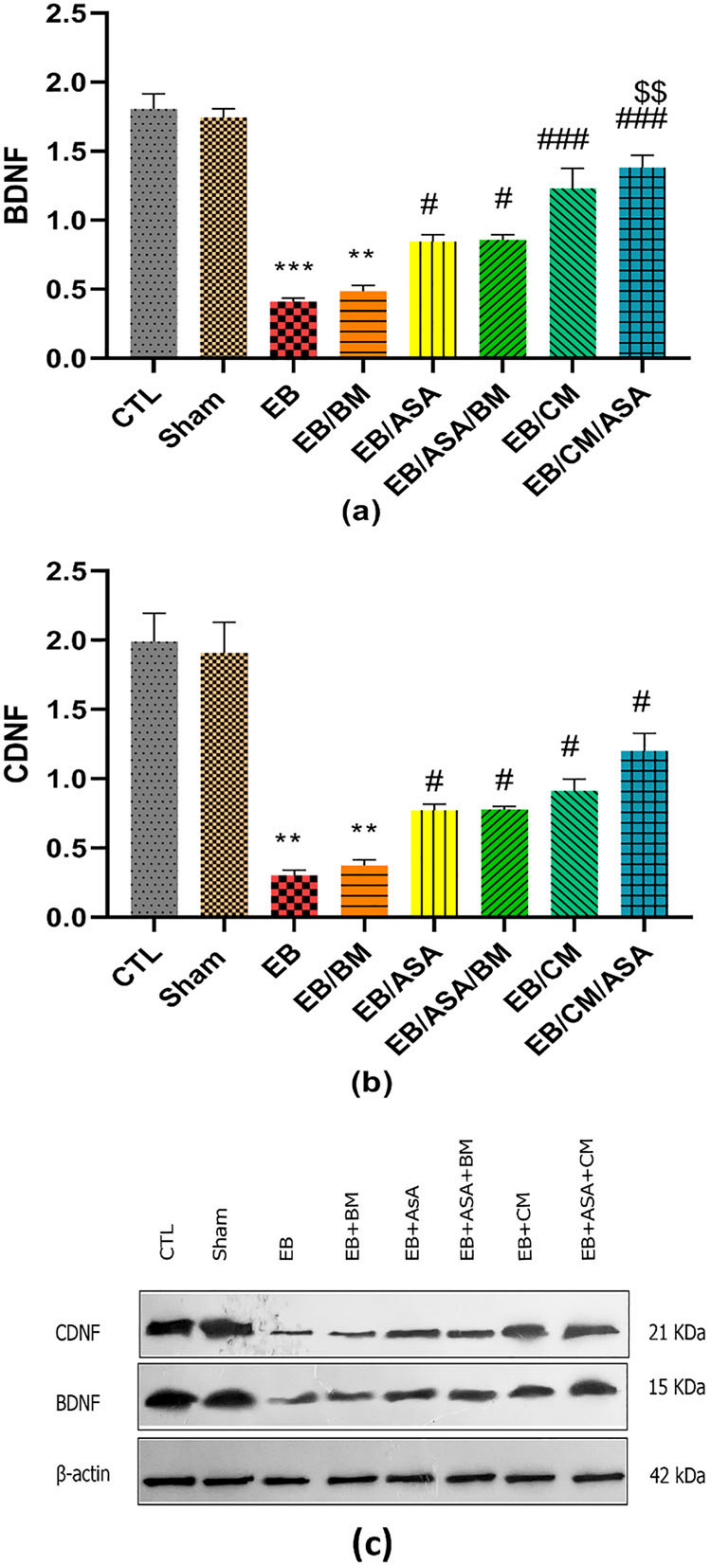
Western blot analysis test assessing the expression of (a) brain‐derived neurotrophic factor (BDNF) and (b) cerebral dopamine neurotrophic factor (CDNF) in the hippocampus. Corresponding bands are represented in (c). Data are expressed as the mean ± standard error of the mean (SEM) (**p < .01, ***p < .001) compared to the control group (#*p* < .05, ###*p* < .001) compared to the ethidium bromide (EB) group and ($$*p* < .01) compared to the EB/acetylsalicylic acid (ASA) group. CTL, control; EB/ASA, ASA‐treated EB group; EB/ASA/BM, BM‐ and ASA‐treated EB group; EB/BM, basal medium (BM)‐treated EB group; EB/CM, mesenchymal stem cell (MSC)–conditioned medium (CM)‐treated EB group; EB/CM/ASA, CM‐ and ASA‐treated EB group; Sham, sham.

Given that the administration of EB is likely to result in damage to the hippocampal regions, we assessed the potential impact of ASA and CM administrations on BDNF and CDNF proteins within the hippocampal tissue samples. EB and EB/BM groups had a decrease in BDNF (Figure 10a) and CDNF (Figure 10b) protein levels in the hippocampus tissue samples compared with the control group. When ASA and/or CM were accompanied with EB, we observed a relative elevated level of BDNF (Figure 10a) and CDNF (Figure 10b) protein levels compared to EB group.

### 3.5 Histological characteristics of the hippocampus

**FIGURE 11:**
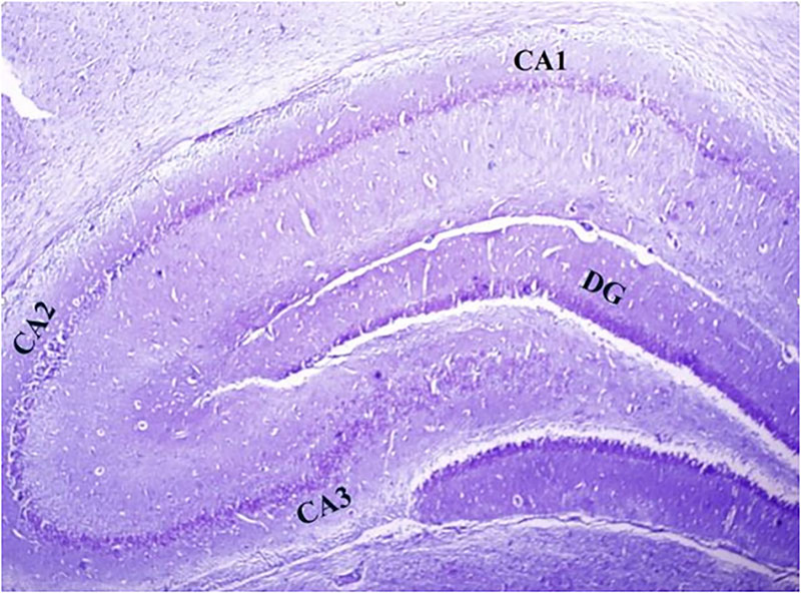
Cresyl violet–stained sections revealed a hippocampus with a C‐shaped structure showing the cornu ammonis (CA) regions and dentate gyrus (DG).

**FIGURE 12:**
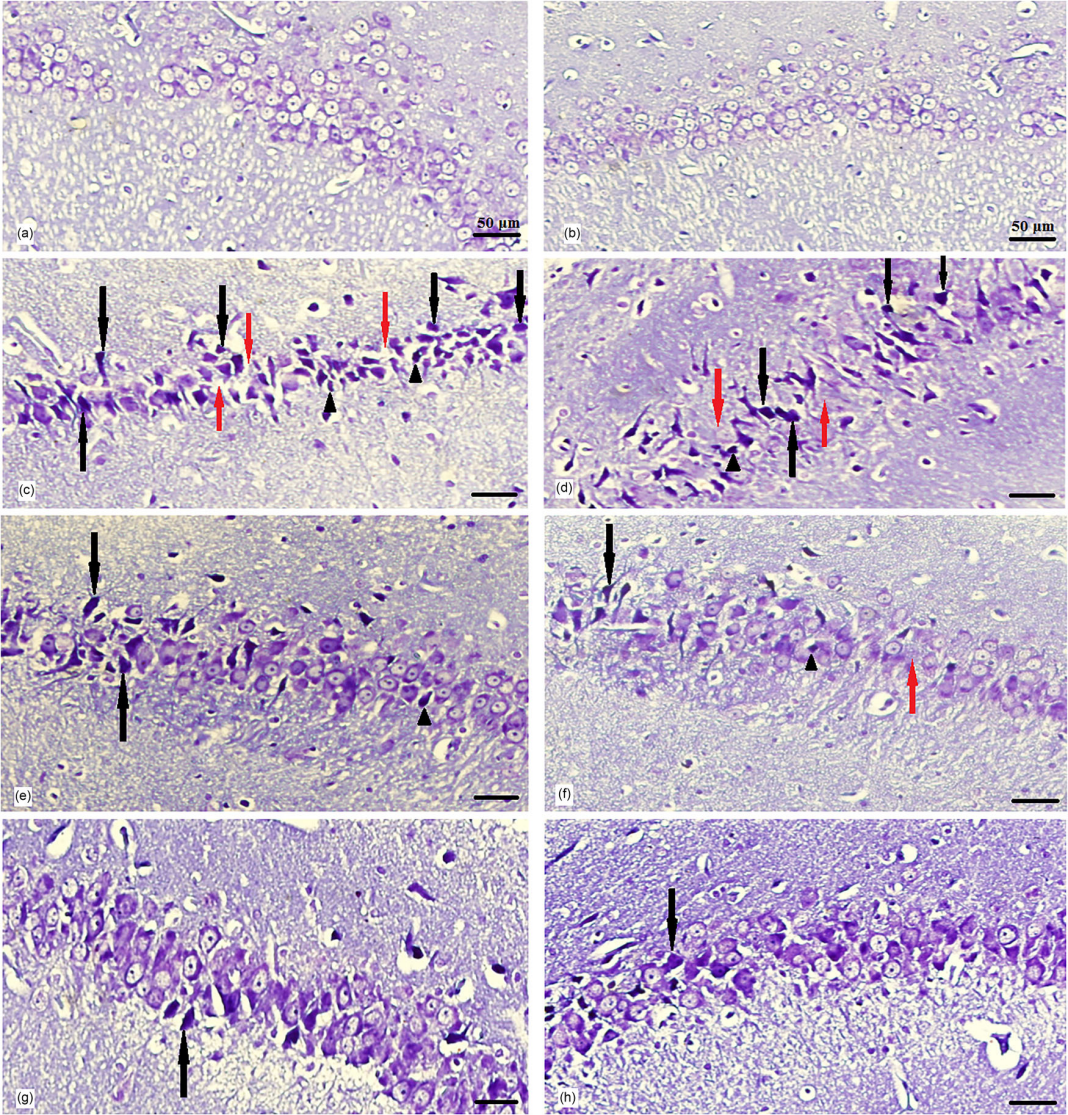
The representative sections stained with Cresyl violet, showing the cornu ammonis region (CA1) in the rat hippocampus for various groups. In these sections, you can observe darkened and shrunken cells (indicated by black arrows), regions with a reduction in cell density (highlighted by red arrows), and nuclei that appear pyknotic (pointed out by arrowheads). (a) Control, (b) sham, (c) ethidium bromide (EB), (d) basal medium (BM)‐treated EB group (EB/BM), (e) acetylsalicylic acid (ASA)‐treated EB group (EB/ASA), (f) BM‐ and ASA‐treated EB group (EB/ASA/BM), (g) mesenchymal stem cell (MSC)‐conditioned medium (CM)‐treated EB group (EB/CM), and (h) CM‐ and ASA‐treated EB group (EB/CM/ASA).

**FIGURE 13:**
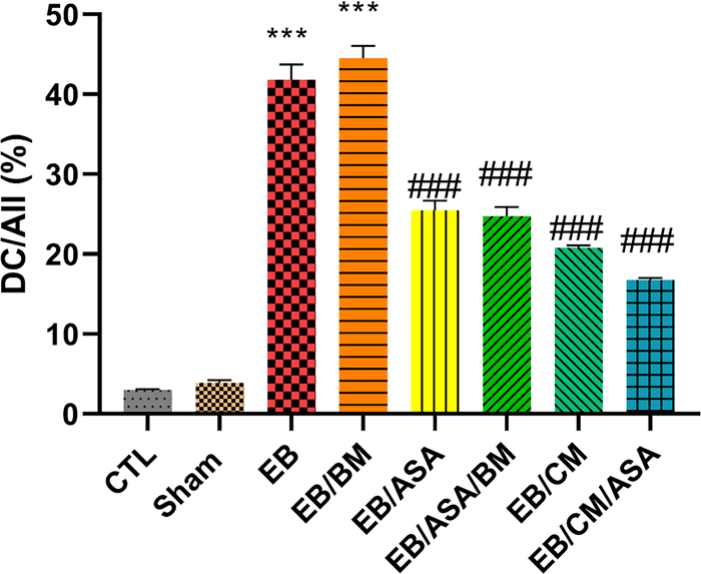
The ratio of degenerated cells (DC) to the total number of cells (All) in the CA1 region of the hippocampus was compared among all experimental groups. Data are expressed as the mean ± standard error of the mean (SEM) (***p < .001) compared to the control group (###*p* < .001) compared to the ethidium bromide (EB) group.

The hippocampus was divided into four subdivisions, namely, CA1, CA2, CA3, and the dentate gyrus (DG), based on the cytoarchitectural characteristics of its principal cells. Under light microscopic examination, Cresyl violet–stained sections of all groups revealed a hippocampus with a C‐shaped structure (Figure 11). The assessment of CA1 area of the hippocampus in experimental groups demonstrated that, in the control group (Figure 12a), CA1 consisted of five to six densely packed layers of small pyramidal cells arranged in a distinct palisade‐like pattern. The pyramidal cells displayed vesicular nuclei, and the cytoplasm of the cells exhibited a pale and basophilic appearance. Examination of the EB and EB/BM groups (Figure 12c,d) revealed significant disorganization, scattering, and a reduction in the number of small pyramidal cells within the CA1 region. These cells appeared darker with hyperchromatic, pyknotic nuclei. Following treatment with ASA or CM, there was a notable improvement in the previously mentioned cell death within the hippocampus. In the Cresyl violet–stained sections of the EB group that received ASA, ASA/BM, and CM (Figure 12e–g), there was a preservation of the smallest pyramidal cells in the CA1 area. Most of these cells exhibited vesicular nuclei, and only a few nuclei appeared pyknotic. The combination therapy of ASA with CM resulted in a significant enhancement in the preservation of pyramidal cells, which exhibited less pronounced shrinkage, and there were only a few apoptotic cells with pyknotic nuclei (Figure 12h). In the Sham group (Figure 12b), histological characteristics were similar to those of the control group, and the small pyramidal cells were organized in a distinctive palisade‐like pattern. These cells had pale basophilic cytoplasm with vesicular nuclei. There was a significant increase in the number of pancellular necrosis pyramidal CA1 cells in both the EB and EB/BM groups compared to the control group (Figure 13). However, EB/ASA, EB/ASA/BM, EB/CM, and EB/CM/ASA groups demonstrated less pancellular necrosis pyramidal CA1 cells compared to the EB group (Figure 13).

## 4 DISCUSSION

Our findings demonstrated that EB administration resulted in cognitive impairments and hippocampal damage in male rats. Accordingly, EB led to deficits in spatial learning and memory, object recognition memory, and passive avoidance learning and memory. These behavioral impairments were accompanied by increased oxidative stress, reduced BDNF and CDNF expression, and pyramidal cell loss in the CA1 region of the hippocampus. The administration of ASA and MSC–CM, either alone or in combination, mitigated the deleterious effects of EB. Treatment with ASA, CM, or their co‐administration improved performance in the behavioral tests, enhanced antioxidative mechanisms, upregulated BDNF and CDNF levels, and attenuated hippocampal neuronal damage compared to EB‐treated rats. Importantly, the combination of ASA and CM resulted in greater neuroprotection than either agent alone in spatial learning and memory, suggesting a synergistic interaction. Overall, the cognitive enhancements observed correlate with the histological preservation of hippocampal pyramidal neurons, particularly in the CA1 region. From the morphological examination of the rat hippocampus, it appears that the cognitive function of EB‐treated rats could be linked to the morphological alterations observed in hippocampal neurons. Furthermore, the application of ASA and CM appears to have a significant mitigating effect on the EB‐induced hippocampal neuronal damage.

In this study, the EB injection method was used to induce hippocampal injury. In order to investigate the relationship between cognitive function and hippocampal neuropathology during the demyelination of neurons in the central nervous system, researchers have turned to experimental models that can induce local damage with minimal invasion, illustrating the processes of neurodegeneration in the brain. Compared to other methods of inducing damage, such as electric current, EB is a compound used to simulate the pathophysiological process of neuron demyelinating diseases and is thus employed in creating experimental models of rodents. It has been suggested that in demyelinating lesions, EB can cause neuroinflammation by activating and multiplying microglia and astrocytes (Rajizadeh et al., 2019). It can also cause demyelination of neurons through neuroinflammation in the hippocampus of rodents, which is more sensitive and vulnerable to threatening and mutagenic stimuli such as EB compared to other areas of the brain (Hollis et al., 2015).

Generally, the ameliorative effects of ASA against EB‐induced neurotoxicity are likely mediated through several complementary mechanisms. ASA possesses anti‐inflammatory properties by inhibiting cyclooxygenase enzymes and prostaglandin production. This helps curb oxidative damage and neural apoptosis after injury. Similar to our observations, in a recent study by Vergil Andrews et al. (2023), it was shown that ASA treatment enhanced novel object recognition as well as spatial learning and memory in experimental aging mice, which was accompanied by increased hippocampal neurogenesis and a decreased number of abnormal microglial cells in the hippocampus. Moreover, consistent with our findings, Patel et al. (2018) reported that aspirin could upregulate the expression of BDNF and other members of the neurotrophin in a mouse model of Alzheimer's disease. Other studies have also pointed out that aspirin could inhibit superoxide anion generation and lipid peroxidation and promote antioxidant signaling pathways, resulting in protection against neuronal damage (Maharaj et al., 2004; Wei et al., 2018).

Meanwhile, CM provides neurotrophic support via paracrine factors secreted by MSCs, including growth factors, cytokines, and extracellular vesicles (Gunawardena et al., 2019). These paracrine signaling molecules can stimulate neurogenesis, synaptic plasticity, anti‐apoptosis, and angiogenesis. This is supported by emerging evidence suggesting CM obtained from adipose tissue–derived MSCs could provide anti‐inflammatory properties against pro‐inflammatory microglia and endothelial cells (Jha et al., 2018) and stabilize neuronal populations through releasing neuroprotective and trophic factors (Szekiova et al., 2018). Consistent with our findings, MSC–CM has also been documented to ameliorate cognitive dysfunction through anti‐inflammatory and antioxidant effects, as well as an increase in BDNF expression (Jiang et al., 2019).

In this study, we observed a synergistic effect of ASA and CM co‐administration on ameliorating EB‐induced spatial memory and learning deficits. Although more studies are needed to provide a definite mechanistic basis for this observation, we conjecture that the synergism between ASA and CM may at least partly arise from ASA altering the sensitivity to growth factors in CM by modulating peroxisome proliferator‐activated receptor alpha (PPARα) signaling. It is demonstrated that PPARα, as a nuclear hormone receptor, is present in the hippocampus and participates in fatty acid metabolism. Findings have suggested that PPARα serves as a novel receptor of ASA for neuroprotection, and the interaction of ASA with PPARα is independent of cyclooxygenase inhibition (Patel et al., 2020). Besides its anti‐coagulation and anti‐inflammatory properties, ASA has been shown to improve amyloid plaque pathology through PPARα signaling (Chandra et al., 2018). Moreover, ASA could bind to PPARα to stimulate hippocampal plasticity and protect against memory detriments (Patel et al., 2018). Based on the existing evidence, probably due to ASA's interaction with PPARα signaling, it can protect against damage in the CA1 area through the influx of calcium and changing the plasticity of the hippocampus (Patel et al., 2018, 2020). More recent findings have revealed the functional role of PPARα in modulating the intrinsic excitability of hippocampal DG neurons and how ASA can regulate DG function through PPARα activation (Xiang et al., 2023). Over the past years, there has been a growing recognition that PPARα may play a role in the molecular mechanisms that regulate the effects of growth factors and cytokines (Biscetti et al., 2008; Inagaki et al., 2007; Moran & Ma, 2015). Hence, it comes as no surprise that the activation of PPARα by ASA could potentially interact with the mechanisms that underlie the effects of CM growth factors, leading to a synergistic impact when ASA and CM are combined. To the best of our knowledge, our study marks the pioneering observation of such an interaction. Further studies are needed to validate this finding and investigate the underlying mechanisms.

**FIGURE 14:**
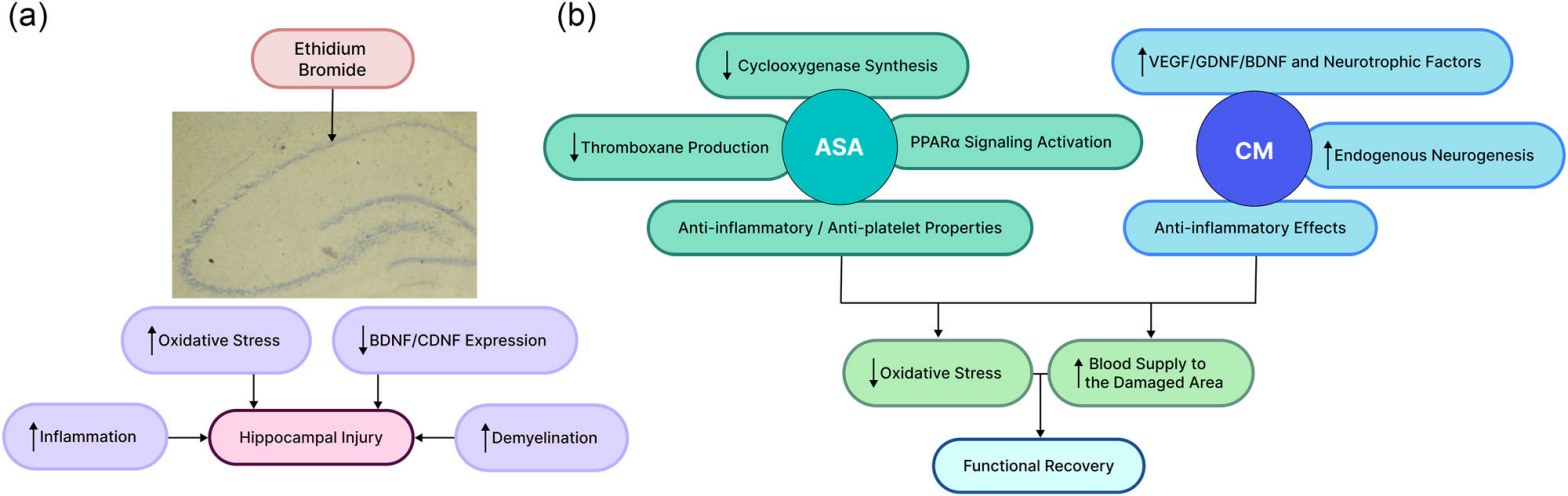
A summary of the effects of the ethidium bromide on the hippocampus (a) and the potential mechanisms underlying the neuroprotective effects of acetylsalicylic acid (ASA) and the culture medium (b). BDNF, brain‐derived neurotrophic factor; CDNF, cerebral dopamine neurotrophic factor; CM, conditioned medium; GDNF, glial cell–derived neurotrophic factor; PPARα, peroxisome proliferator–activated receptor alpha; VEGF, vascular endothelial growth factor.

Generally, compared to the injection of stem cells through cell therapy, the injection of CM seems to be a safer model. It is known that when cells are injected into animals, some become trapped in tissues such as the liver, lungs, and other internal organs. Intravenous injection of cells results in fewer cells reaching the brain tissue and, ultimately, the damaged area (Shamsara et al., 2018; Sheibani et al., 2015). However, the injection of CM derived from cells, while containing several types of neurotrophic factors for injury recovery, neither stimulates the immune system nor causes local vascular occlusions (Zhang et al., 2022). Therefore, the combination of ASA and CM might provide clinical therapeutic utility after more comprehensive research. However, to facilitate clinical translation, the long‐term efficacy of these agents is needed to be more comprehensively assessed. Taken together, our preliminary results suggest that the combination of ASA and CM offers neuroprotection against hippocampal injury. Our behavioral outcomes are consistent with the histopathological and molecular findings. However, the exact mechanism of the effect of ASA and CM in preventing neuronal degeneration requires further research. Based on the existing literature, we provided a schematic summary of the potential underlying mechanisms (Figure 14).

Some limitations should be noted. First, only male rats were utilized in this study, so sex differences remain unexplored. Second, behavioral assessments were conducted shortly after EB injection, so whether ASA and CM confer long‐term cognition preservation requires further investigation. Furthermore, in this study, fixed doses of agents were used based on prior research. We did not explore a range of doses, which might have provided a more comprehensive understanding of the optimal therapeutic regimen. Future studies should investigate different doses and combinations to identify the most effective and potentially synergistic concentrations of ASA and CM. Moreover, further in‐depth mechanistic analyses may help clarify the precise signaling pathways underlying the combined impact of ASA and CM. Future studies should focus on these issues and assess the translational potential of ASA and CM therapy for neurodegenerative disorders affecting the hippocampus.

## 5 CONCLUSIONS

Overall, this study demonstrates that ASA combined with CM from adipose‐derived MSCs provides amelioration of EB‐induced hippocampal damage and cognitive decline in male rats. Our findings provide preliminary evidence of the potential synergistic effects of ASA and CM, which warrants further investigations.

## AUTHOR CONTRIBUTIONS

**Iman Zangiabadi**: Conceptualization; investigation; writing—original draft; formal analysis; software; data curation. **Mehran Ilaghi**: Writing—original draft; visualization; writing—review and editing; conceptualization; investigation. **Ali Shamsara**: Conceptualization; investigation; methodology; supervision; project administration; writing—review and editing. **Seyed Hassan Eftekhar‐Vaghefi**: Conceptualization; investigation; validation; supervision. **Mona Saheli**: Conceptualization; investigation; validation; supervision. **Mohammad Shabani**: Conceptualization; validation; formal analysis; supervision; writing—review and editing.

## CONFLICT OF INTEREST STATEMENT

The authors declare no conflicts of interest.

### PEER REVIEW

The peer review history for this article is available at https://publons.com/publon/10.1002/brb3.70010.

## CONSENT FOR PUBLICATION

Not applicable.

## Data Availability

The datasets used or analyzed during the current study are available from the corresponding author upon request.

## References

[brb370010-bib-0001] Abolhasani, F. , Pourshojaei, Y. , Mohammadi, F. , Esmaeilpour, K. , Asadipour, A. , Ilaghi, M. , & Shabani, M. (2023). Exploring the potential of a novel phenoxyethyl piperidine derivative with cholinesterase inhibitory properties as a treatment for dementia: Insights from STZ animal model of dementia. Neuroscience Letters, 810, 137332. 10.1016/j.neulet.2023.137332 37302565

[brb370010-bib-0002] Aghaei, Z. , Karbalaei, N. , Namavar, M. R. , Haghani, M. , Razmkhah, M. , Ghaffari, M. K. , & Nemati, M. (2023). Neuroprotective effect of Wharton's jelly‐derived mesenchymal stem cell‐conditioned medium (WJMSC‐CM) on diabetes‐associated cognitive impairment by improving oxidative stress, neuroinflammation, and apoptosis. Stem Cells International, 2023, 7852394. 10.1155/2023/7852394 37081849 PMC10113062

[brb370010-bib-0003] Akhondzadeh, F. , Kadkhodaee, M. , Seifi, B. , Ashabi, G. , Kianian, F. , Abdolmohammadi, K. , Izad, M. , Adelipour, M. , & Ranjbaran, M. (2020). Adipose‐derived mesenchymal stem cells and conditioned medium attenuate the memory retrieval impairment during sepsis in rats. Molecular Neurobiology, 57, 3633–3645. 10.1007/s12035-020-01991-6 32562236

[brb370010-bib-0004] Asadi‐Shekaari, M. , Vaghefi, H. E. , Abadi pour, M. E. , Sheibani, V. , Shams Ara, A. , & Behbahani, P. (2011). Antiapoptotic effects of aspirin on CA1 pyramidal neurons in adult rats. Iranian Journal of Pathology, 6(4), 187–192.

[brb370010-bib-0005] Bejeshk, M. A. , Aminizadeh, A. H. , Jafari, E. , Motamedi, S. , Zangiabadi, I. , Ghasemi, A. , Fathi, M. , Nezhadi, A. , Akhgarandouz, F. , Bejeshk, F. , Mohammadi, L. , Mohammadi, F. , & Rajizadeh, M. A. (2023). Myrtenol ameliorates recognition memories’ impairment and anxiety‐like behaviors induced by asthma by mitigating hippocampal inflammation and oxidative stress in rats. Neuroimmunomodulation, 30(1), 42–54. 10.1159/000528626 36657415

[brb370010-bib-0006] Bejeshk, M. A. , Beik, A. , Aminizadeh, A. H. , Salimi, F. , Bagheri, F. , Sahebazzamani, M. , Najafipour, H. , & Rajizadeh, M. A. (2023). Perillyl alcohol (PA) mitigates inflammatory, oxidative, and histopathological consequences of allergic asthma in rats. Naunyn‐Schmiedeberg's Archives of Pharmacology, 396(6), 1235–1245. 10.1007/s00210-023-02398-5 36707429

[brb370010-bib-0007] Biscetti, F. , Gaetani, E. , Flex, A. , Aprahamian, T. , Hopkins, T. , Straface, G. , Pecorini, G. , Stigliano, E. , Smith, R. C. , Angelini, F. , Castellot, J. J. , & Pola, R. (2008). Selective activation of peroxisome proliferator–activated receptor (PPAR) α and PPARγ induces neoangiogenesis through a vascular endothelial growth factor–dependent mechanism. Diabetes, 57(5), 1394–1404. 10.2337/db07-0765 18268046

[brb370010-bib-0008] Chandra, S. , Jana, M. , & Pahan, K. (2018). Aspirin induces lysosomal biogenesis and attenuates amyloid plaque pathology in a mouse model of Alzheimer's disease via PPARα. Journal of Neuroscience, 38(30), 6682–6699. 10.1523/JNEUROSCI.0054-18.2018 29967008 PMC6067079

[brb370010-bib-0009] De Cristóbal, J. , Moro, M. A. , Dávalos, A. , Castillo, J. , Leza, J. C. , Camarero, J. , Colado, M. I. , Lorenzo, P. , & Lizasoain, I. (2001). Neuroprotective effect of aspirin by inhibition of glutamate release after permanent focal cerebral ischaemia in rats. Journal of Neurochemistry, 79(2), 456–459. 10.1046/j.1471-4159.2001.00600.x 11677274

[brb370010-bib-0010] de Ugarte, D. A. , Morizono, K. , Elbarbary, A. , Alfonso, Z. , Zuk, P. A. , Zhu, M. , Dragoo, J. L. , Ashjian, P. , Thomas, B. , Benhaim, P. , Chen, I. , Fraser, J. , & Hedrick, M. H. (2003). Comparison of multi‐lineage cells from human adipose tissue and bone marrow. Cells Tissues Organs, 174(3), 101–109. 12835573 10.1159/000071150

[brb370010-bib-0012] Eftekhar‐Vaghefi, S. H. , Zahmatkesh, L. , Salehinejad, P. , Totonchi, S. , & Shams‐Ara, A. (2015). Evaluation of neurogenic potential of human umbilical cord mesenchymal cells; a time‐and concentration‐dependent manner. Iranian Biomedical Journal, 19(2), 82. 25864812 10.6091/ibj.1452.2015 PMC4412918

[brb370010-bib-0013] Egashira, Y. , Sugitani, S. , Suzuki, Y. , Mishiro, K. , Tsuruma, K. , Shimazawa, M. , Yoshimura, S. , Iwama, T. , & Hara, H. (2012). The conditioned medium of murine and human adipose‐derived stem cells exerts neuroprotective effects against experimental stroke model. Brain Research, 1461, 87–95. 10.1016/j.brainres.2012.04.033 22608076

[brb370010-bib-0014] Esmaeilpour, K. , Jafari, E., Rostamabadi, F., Khaleghi, M., Akhgarandouz, F., Hosseini, M., Najafipour, H., Khodadoust, M., Sheibani, V., Rajizadeh, M.A.(2023). Myrtenol inhalation mitigates asthma‐induced cognitive impairments: An electrophysiological, behavioral, histological, and molecular study. Molecular Neurobiology, 61(7), 4891–4907. 38148370 10.1007/s12035-023-03863-1

[brb370010-bib-0015] Guillén, M. I. , Platas, J. , Pérez Del Caz, M. D. , Mirabet, V. , & Alcaraz, M. J. (2018). Paracrine anti‐inflammatory effects of adipose tissue‐derived mesenchymal stem cells in human monocytes. Frontiers in Physiology, 9, 661. 10.3389/fphys.2018.00661 29904354 PMC5990614

[brb370010-bib-0016] Gunawardena, T. N. A. , Rahman, M. T. , Abdullah, B. J. J. , & Abu Kasim, N. H. (2019). Conditioned media derived from mesenchymal stem cell cultures: The next generation for regenerative medicine. Journal of Tissue Engineering and Regenerative Medicine, 13(4), 569–586. 10.1002/term.2806 30644175

[brb370010-bib-0017] Han, Y. U. , Li, X. , Zhang, Y. , Han, Y. , Chang, F. , & Ding, J. (2019). Mesenchymal stem cells for regenerative medicine. Cells, 8(8), 886. 10.3390/cells8080886 31412678 PMC6721852

[brb370010-bib-0018] Hanie, M. H. , Mohammad Reza, A. , Mansoureh, S. , Fatemeh, S. B. , & Ali, S. (2024). Exploring the impact of melatonin and omega‐3, individually and in combination, on cognitive function, histological changes, and oxidant‐antioxidant balance in male rats with dorsal CA1 hippocampal lesions. Brain Research, 1840, 149046. 10.1016/j.brainres.2024.149046 38821333

[brb370010-bib-0019] Hollis, E. R. , Ishiko, N. , Tolentino, K. , Doherty, E. , Rodriguez, M. J. , Calcutt, N. A. , & Zou, Y. (2015). A novel and robust conditioning lesion induced by ethidium bromide. Experimental neurology, 265, 30–39. 10.1016/j.expneurol.2014.12.004 25541322 PMC4346483

[brb370010-bib-0020] Inagaki, T. , Dutchak, P. , Zhao, G. , Ding, X. , Gautron, L. , Parameswara, V. , Li, Y. , Goetz, R. , Mohammadi, M. , Esser, V. , Elmquist, J. K. , Gerard, R. D. , Burgess, S. C. , Hammer, R. E. , Mangelsdorf, D. J. , & Kliewer, S. A. (2007). Endocrine regulation of the fasting response by PPARα‐mediated induction of fibroblast growth factor 21. Cell Metabolism, 5(6), 415–425. 10.1016/j.cmet.2007.05.003 17550777

[brb370010-bib-0021] Ivanova‐Todorova, E. , Bochev, I. , Dimitrov, R. , Belemezova, K. , Mourdjeva, M. , Kyurkchiev, S. , Kinov, P. , Altankova, I. , & Kyurkchiev, D. (2012). Conditioned medium from adipose tissue‐derived mesenchymal stem cells induces CD_4_+ FOXP_3_+ cells and increases IL‐10 secretion. BioMed Research International, 2012, 295167. 10.1155/2012/295167 PMC3521492 23251077

[brb370010-bib-0022] Jha, K. A. , Pentecost, M. , Lenin, R. , Klaic, L. , Elshaer, S. L. , Gentry, J. , Russell, J. M. , Beland, A. , Reiner, A. , Jotterand, V. , Sohl, N. , & Gangaraju, R. (2018). Concentrated conditioned media from adipose tissue derived mesenchymal stem cells mitigates visual deficits and retinal inflammation following mild traumatic brain injury. International Journal of Molecular Sciences, 19(7), 2016. 10.3390/ijms19072016 29997321 PMC6073664

[brb370010-bib-0023] Jiang, Y. E. , Gao, H. , Yuan, H. , Xu, H. , Tian, M. , Du, G. , & Xie, W. (2019). Amelioration of postoperative cognitive dysfunction in mice by mesenchymal stem cell‐conditioned medium treatments is associated with reduced inflammation, oxidative stress and increased BDNF expression in brain tissues. Neuroscience Letters, 709, 134372. 10.1016/j.neulet.2019.134372 31295540

[brb370010-bib-0024] Krawczenko, A. , & Klimczak, A. (2022). Adipose tissue‐derived mesenchymal stem/stromal cells and their contribution to angiogenic processes in tissue regeneration. International Journal of Molecular Sciences, 23(5), 2425. 10.3390/ijms23052425 35269568 PMC8910401

[brb370010-bib-0025] Leuner, B. , & Gould, E. (2010). Structural plasticity and hippocampal function. Annual Review of Psychology, 61, 111–140. 10.1146/annurev.psych.093008.100359 PMC3012424 19575621

[brb370010-bib-0026] Ma, L. , Cui, X.‐L. , Wang, Y. , Li, X.‐W. , Yang, F. , Wei, D. , & Jiang, W. (2012). Aspirin attenuates spontaneous recurrent seizures and inhibits hippocampal neuronal loss, mossy fiber sprouting and aberrant neurogenesis following pilocarpine‐induced status epilepticus in rats. Brain Research, 1469, 103–113. 10.1016/j.brainres.2012.05.058 22765917

[brb370010-bib-0027] Maharaj, D. S. , Saravanan, K. S. , Maharaj, H. , Mohanakumar, K. P. , & Daya, S. (2004). Acetaminophen and aspirin inhibit superoxide anion generation and lipid peroxidation, and protect against 1‐methyl‐4‐phenyl pyridinium‐induced dopaminergic neurotoxicity in rats. Neurochemistry International, 44(5), 355–360. 10.1016/S0197-0186(03)00170-0 14643753

[brb370010-bib-0028] Mehrabadi, S. , Motevaseli, E. , Sadr, S. S. , & Moradbeygi, K. (2020). Hypoxic‐conditioned medium from adipose tissue mesenchymal stem cells improved neuroinflammation through alternation of toll like receptor (TLR) 2 and TLR_4_ expression in model of Alzheimer's disease rats. Behavioural Brain Research, 379, 112362. 10.1016/j.bbr.2019.112362 31739000

[brb370010-bib-0029] Mehrabani, M. , Mohammadyar, S. , Rajizadeh, M. A. , Bejeshk, M. A. , Ahmadi, B. , Nematollahi, M. H. , Mirtajaddini Goki, M. , Bahrampour Juybari, K. , & Amirkhosravi, A. (2023). Boosting therapeutic efficacy of mesenchymal stem cells in pulmonary fibrosis: The role of genetic modification and preconditioning strategies. Iranian Journal of Basic Medical Sciences, 26(9), 1001. 37605719 10.22038/IJBMS.2023.69023.15049 PMC10440137

[brb370010-bib-0030] Moran, E. P. , & Ma, J.‐X. (2015). Therapeutic effects of PPARα on neuronal death and microvascular impairment. PPAR Research, 2015, 595426. 10.1155/2015/595426 25705219 PMC4326216

[brb370010-bib-0031] Nakagami, H. , Maeda, K. , Morishita, R. , Iguchi, S. , Nishikawa, T. , Takami, Y. , Kikuchi, Y. , Saito, Y. , Tamai, K. , Ogihara, T. , & Kaneda, Y. (2005). Novel autologous cell therapy in ischemic limb disease through growth factor secretion by cultured adipose tissue‐derived stromal cells. Arteriosclerosis, Thrombosis, and Vascular Biology, 25(12), 2542–2547. 10.1161/01.ATV.0000190701.92007.6d 16224047

[brb370010-bib-0032] Patel, D. , Roy, A. , Kundu, M. , Jana, M. , Luan, C.‐H. , Gonzalez, F. J. , & Pahan, K. (2018). Aspirin binds to PPARα to stimulate hippocampal plasticity and protect memory. Proceedings of the National Academy of Sciences of the United States of America, 115(31), E7408–E7417. 10.1073/pnas.1802021115 30012602 PMC6077698

[brb370010-bib-0033] Patel, D. , Roy, A. , & Pahan, K. (2020). PPARα serves as a new receptor of aspirin for neuroprotection. Journal of Neuroscience Research, 98(4), 626–631. 10.1002/jnr.24561 31797405 PMC7015783

[brb370010-bib-0011] Pour, J. D. M. , Hosseinmardi, N. , Janahmadi, M. , Fathollahi, Y. , Motamedi, F. , & Hooshmandi, M. (2013). Aspirin changes short term synaptic plasticity in CA1 area of the rat hippocampus. Physiology and Pharmacology, 17(3), 298–307.

[brb370010-bib-0034] Rajizadeh, M. A. , Esmaeilpour, K. , Masoumi‐Ardakani, Y. , Bejeshk, M. A. , Shabani, M. , Nakhaee, N. , Ranjbar, M. P. , Borzadaran, F. M. , & Sheibani, V. (2018). Voluntary exercise impact on cognitive impairments in sleep‐deprived intact female rats. Physiology & Behavior, 188, 58–66. 29360489 10.1016/j.physbeh.2017.12.030

[brb370010-bib-0035] Rajizadeh, M. A. , Esmaeilpour, K. , Motamedy, S. , Mohtashami Borzadaranb, F. , & Sheibani, V. (2020). Voluntary exercise modulates learning & memory and synaptic plasticity impairments in sleep deprived female rats. Brain Research, 1729, 146598. 31866363 10.1016/j.brainres.2019.146598

[brb370010-bib-0036] Rajizadeh, M. A. , Khaksari, M. , Bejeshk, M. A. , Amirkhosravi, L. , Jafari, E. , Jamalpoor, Z. , & Nezhadi, A. (2023). The role of inhaled estradiol and myrtenol, alone and in combination, in modulating behavioral and functional outcomes following traumatic experimental brain injury: Hemodynamic, molecular, histological and behavioral study. Neurocritical Care, 39(2), 478–498. 10.1007/s12028-023-01720-6 37100976

[brb370010-bib-0037] Rajizadeh, M. A. , Sheibani, V. , Bejeshk, M. A. , Mohtashami Borzadaran, F. , Saghari, H. , & Esmaeilpour, K. (2019). The effects of high intensity exercise on learning and memory impairments followed by combination of sleep deprivation and demyelination induced by ethidium bromide. International Journal of Neuroscience, 129(12), 1166–1178. 10.1080/00207454.2019.1640695 31274046

[brb370010-bib-0038] Ramalingam, M. , Jeong, H.‐S. , Hwang, J. , Cho, H.‐H. , Kim, B. C. , Kim, E. , & Jang, S. (2022). Autophagy signaling by neural‐induced human adipose tissue‐derived stem cell‐conditioned medium during rotenone‐induced toxicity in SH‐SY5Y cells. International Journal of Molecular Sciences, 23(8), 4193. 10.3390/ijms23084193 35457010 PMC9031864

[brb370010-bib-0039] Shabani, M. , Ilaghi, M. , Naderi, R. , & Razavinasab, M. (2023). The hyperexcitability of laterodorsal tegmentum cholinergic neurons accompanies adverse behavioral and cognitive outcomes of prenatal stress. Scientific Reports, 13(1), 6011. 10.1038/s41598-023-33016-2 37045899 PMC10097720

[brb370010-bib-0040] Shahraki, S. , Esmaeilpour, K. , Shabani, M. , Sepehri, G. , Rajizadeh, M. A. , Maneshian, M. , Joushi, S. , & Sheibani, V. (2022). Choline chloride modulates learning, memory, and synaptic plasticity impairments in maternally separated adolescent male rats. International Journal of Developmental Neuroscience, 82(1), 19–38. 10.1002/jdn.10155 34727391

[brb370010-bib-0041] Shamsara, A. , Sheibani, V. , Asadi‐Shekaari, M. , & Nematollahi‐Mahani, S. N. (2018). Neural like cells and acetyl‐salicylic acid alter rat brain structure and function following transient middle cerebral artery occlusion. Biomolecular Concepts, 9(1), 155–168. 10.1515/bmc-2018-0014 30864349

[brb370010-bib-0042] Sheibani, V. , Sheibani, V. , Esmaeilpour, K. , Eslaminejad, T. , & Nematollahi‐Mahani, S. N. (2015). Coadministration of the human umbilical cord matrix‐derived mesenchymal cells and aspirin alters postischemic brain injury in rats. Journal of Stroke and Cerebrovascular Diseases, 24(9), 2005–2016. 26145764 10.1016/j.jstrokecerebrovasdis.2015.04.049

[brb370010-bib-0043] Shin, H. , Ryu, H. H. , Kwon, O. , Park, B.‐S. , & Jo, S. J. (2015). Clinical use of conditioned media of adipose tissue‐derived stem cells in female pattern hair loss: A retrospective case series study. International Journal of Dermatology, 54(6), 730–735. 10.1111/ijd.12650 25777970

[brb370010-bib-0044] Shree, N. , & Bhonde, R. R. (2017). Conditioned media from adipose tissue derived mesenchymal stem cells reverse insulin resistance in cellular models. Journal of Cellular Biochemistry, 118(8), 2037–2043. 10.1002/jcb.25777 27791278

[brb370010-bib-0045] Stojanović, S. , & Najman, S. (2019). The effect of conditioned media of stem cells derived from lipoma and adipose tissue on macrophages’ response and wound healing in indirect co‐culture system in vitro. International Journal of Molecular Sciences, 20(7), 1671. 10.3390/ijms20071671 30987193 PMC6479913

[brb370010-bib-0046] Szekiova, E. , Slovinska, L. , Blasko, J. , Plsikova, J. , & Cizkova, D. (2018). The neuroprotective effect of rat adipose tissue‐derived mesenchymal stem cell‐conditioned medium on cortical neurons using an in vitro model of SCI inflammation. Neurological Research, 40(4), 258–267. 10.1080/01616412.2018.1432266 29384015

[brb370010-bib-0047] Tsai, M.‐J. , Liou, D.‐Y. , Lin, Y.‐R. , Weng, C.‐F. , Huang, M.‐C. , Huang, W.‐C. , Tseng, F.‐W. , & Cheng, H. (2018). Attenuating spinal cord injury by conditioned medium from bone marrow mesenchymal stem cells. Journal of Clinical Medicine, 8(1), 23. 10.3390/jcm8010023 30585207 PMC6352201

[brb370010-bib-0048] Turner, V. S. , O'sullivan, R. O. , & Kheirbek, M. A. (2022). Linking external stimuli with internal drives: A role for the ventral hippocampus. Current Opinion in Neurobiology, 76, 102590. 10.1016/j.conb.2022.102590 35753108 PMC9818033

[brb370010-bib-0049] Vergil Andrews, J. F. , Selvaraj, D. B. , Kumar, A. , Roshan, S. A. , Anusuyadevi, M. , & Kandasamy, M. (2023). A mild dose of aspirin promotes hippocampal neurogenesis and working memory in experimental ageing mice. Brain Sciences, 13(7), 1108. 10.3390/brainsci13071108 37509038 PMC10376986

[brb370010-bib-0050] Watson, C. (2007). The rat brain in stereotaxic coordinates: Hard cover edition. Academic Press.

[brb370010-bib-0051] Wei, W. , Shurui, C. , Zipeng, Z. , Hongliang, D. , Hongyu, W. , Yuanlong, L. , Kang, Z. , Zhaoliang, S. , Yue, G. , Chang, L. , & Mei, X. (2018). Aspirin suppresses neuronal apoptosis, reduces tissue inflammation, and restrains astrocyte activation by activating the Nrf2/HO‐1 signaling pathway. NeuroReport, 29(7), 524–531. 10.1097/WNR.0000000000000969 29381509

[brb370010-bib-0052] Xiang, G. , Liu, X. , Wang, J. , Lu, S. , Yu, M. , Zhang, Y. , Sun, B. , Huang, B. , Lu, X.‐Y. , Li, X. , & Zhang, D. (2023). Peroxisome proliferator‐activated receptor‐α activation facilitates contextual fear extinction and modulates intrinsic excitability of dentate gyrus neurons. Translational Psychiatry, 13(1), 206. 10.1038/s41398-023-02496-1 37322045 PMC10272150

[brb370010-bib-0053] Zangiabadi, I. , Afarinesh, M. R. , Shamsara, A. , & Eftekhar‐Vaghefi, S. H. (2019). Movento effects on learning and hippocampal brain‐derived neurotrophic factor protein of adult male rats. Environmental Science and Pollution Research, 26, 36615–36622. 10.1007/s11356-019-06809-0 31734837

[brb370010-bib-0054] Zare, D. , Rajizadeh, M. A. , Maneshian, M. , Jonaidi, H. , Sheibani, V. , Asadi‐Shekaari, M. , Yousefi, M. , & Esmaeilpour, K. (2021). Inhibition of protease‐activated receptor 1 (PAR1) ameliorates cognitive performance and synaptic plasticity impairments in animal model of Alzheimer's diseases. Psychopharmacology, 238, 1645–1656. 10.1007/s00213-021-05798-8 33624157

[brb370010-bib-0055] Zarin, M. , Karbalaei, N. , Keshtgar, S. , & Nemati, M. (2019). Platelet‐rich plasma improves impaired glucose hemostasis, disrupted insulin secretion, and pancreatic oxidative stress in streptozotocin‐induced diabetic rat. Growth Factors, 37(5–6), 226–237. 10.1080/08977194.2020.1735382 32151173

[brb370010-bib-0056] Zhang, B. , Wu, Y. , Mori, M. , & Yoshimura, K. (2022). Adipose‐derived stem cell conditioned medium and wound healing: A systematic review. Tissue Engineering Part B: Reviews, 28(4), 830–847. 10.1089/ten.teb.2021.0100 34409890

